# Female intuition in economics and conscious leadership: a comprehensive integrative review of conceptual foundations, cultural moderators, and future directions

**DOI:** 10.3389/fsoc.2025.1560090

**Published:** 2025-09-30

**Authors:** Roberto Gómez Tobías

**Affiliations:** Tecnológico de Monterrey, Monterrey, Mexico

**Keywords:** female intuition, conscious leadership, behavioral economics, cognitive neuroscience, feminist economics, ethical decision-making, cultural and institutional moderators, risk perception

## Abstract

**Background:**

The notion of female intuition has long been marginalized or misconstrued within economic and leadership discourse. Recent contributions from cognitive neuroscience, feminist economics, and organizational theory offer new insights into intuition as a nuanced form of embodied judgment, responsive to both ethical and contextual demands. In response to this gap, the present review systematically examines how this epistemic capacity is conceptualized, empirically explored, and shaped by institutional and cultural conditions.

**Objectives:**

Building on this foundation, this analysis articulates four interrelated objectives: (1) To define and integrate the construct of female intuition within economic and leadership domains; (2) To assess its influence on innovation, risk perception, and decision quality; (3) To explore cultural, structural, and neuropsychological moderators of its expression and legitimacy; (4) To propose a multidimensional framework grounded in feminist and neuroconstructivist epistemologies.

**Methods:**

A systematic and integrative protocol was applied to peer-reviewed literature published between 2000 and 2024 across Scopus, Web of Science, PsycINFO, PubMed, and EconLit. Boolean operators and controlled vocabulary (e.g., MeSH terms, APA Thesaurus descriptors) were used to ensure precision. Eligible studies addressed intuition, leadership, or decision-making through gendered lens. Data extraction included metadata (authorship, year, country), methodology, theoretical framing, and findings. Quality appraisal followed CASP (for qualitative designs), MMAT 2018 (for mixed methods), and AMSTAR 2 (for reviews). Thematic synthesis was complemented by bibliometric mapping using VOSviewer to identify citation clusters and emerging research fronts.

**Discussion:**

By confronting epistemic biases and expanding the discourse on intuitive cognition, this review contributes to a more inclusive understanding of leadership intelligence. Its findings offer theoretical grounding for future research, inform gender-sensitive educational practices, and support the design of economic policies that recognize cognitive diversity as a strategic asset.

**Systematic review registration:**

Identifier: doi: 10.17605/OSF.IO/TR5DP.

## 1 Introduction

In an era defined by volatility and systemic complexity, the capacity for sound judgment under uncertainty has become a cornerstone of effective leadership (Kniffin et al., 2021; Luoma and Martela, 2021). Despite this shift, conventional decision-making frameworks—rooted in rational choice theory—continue to dominate organizational and economic models, even as mounting evidence shows they overlook the emotional, relational, and context-dependent dimensions required to navigate ambiguity (Luoma and Martela, 2021). These paradigms often reproduce masculinized norms of authority, marginalizing embodied or intuitive ways of knowing, particularly those associated with women’s cognition (Eagly and Carli, 2007; Sinclair, 2011b).

Across fields such as behavioral economics, neuroleadership, and cognitive psychology, intuition is increasingly understood as a fast, adaptive form of reasoning activated under pressure (Adinolfi and Loia, 2022). However, its gendered dimensions remain significantly undertheorized. Female intuition—defined here as the integration of emotional intelligence, contextual sensitivity, embodied insight, and ethical reflexivity—has frequently been dismissed as irrational or anecdotal, rather than recognized as a complex cognitive process shaped by sociocultural scripts and institutional constraints (Fine, 2017a; Salazar Montoya and Kew, 2023; Sinclair, 2011a). Even as advances in embodied cognition and neuroconstructivist theory offer robust analytical tools, most frameworks continue to treat intuition as gender-neutral or implicitly masculine (Hodgkinson and Sadler-Smith, 2017; Isenman, 2013), neglecting the distinctive ways in which women engage with uncertainty, ambiguity, and relational complexity in leadership contexts.

This historical marginalization aligns with Gigerenzer’s (2023) argument that intuition was sidelined in scientific discourse precisely because it was perceived as inherently feminine in a masculinized rational order. Our review builds on this paradox: although intuition has been feminized in cultural narratives and thus devalued, it has simultaneously been redefined in analytical terms that masculinize its legitimacy. This dual dynamic—discursive exclusion and methodological assimilation—reinforces the epistemic tension surrounding intuitive cognition.

Preliminary bibliometric mapping reinforces this theoretical blind spot: among over 1,750 articles referencing “intuition” and “leadership” (2000–2024), fewer than 5% explicitly engage gender, and less than 1% theorize female intuition as a distinct construct. The majority either universalize intuition or treat it as an acultural trait, ignoring its embeddedness in gender roles, institutional logics, and power asymmetries.

This review addresses that gap through a systematic and integrative synthesis of 142 peer-reviewed studies across five continents and disciplinary domains, including economics, cognitive neuroscience, gender studies, and organizational behavior. Following PRISMA 2020 standards and preregistered on the Open Science Framework (OSF), the review applies three validated quality appraisal tools (AMSTAR 2, MMAT, CASP) and triangulates thematic synthesis with bibliometric analysis.

Female intuition is herein defined as a context-sensitive, somatically grounded, and ethically attuned cognitive mode (Fine, 2017a; Sinclair, 2011a). This review reframes it as a situated epistemic capacity—one shaped by gendered socialization, neurocognitive integration, and institutional affordances (Isenman, 2009a; Salazar Montoya and Kew, 2023). Crucially, intuition is not presented as an essentialist or biologically determined trait exclusive to women, but rather as a strategic analytical construct whose expression and legitimacy are modulated by identity and context. In this sense, the term “female intuition” functions analytically—not biologically—reclaiming a historically marginalized form of leadership intelligence.

This analysis pursues four interrelated aims:

1. To articulate a precise, operational definition of female intuition, distinguishing it from general intuition or affective tendencies;
2. To synthesize empirical evidence on its influence in leadership effectiveness, decision quality, innovation, and risk perception;
3. To examine cultural, structural, and neuropsychological moderators that shape its expression, legitimacy, and strategic utility;
4. To propose an integrative theoretical framework—drawing from dual-process theory, feminist organizational epistemology, and embodied cognition—to inform future research and practice.

By addressing these aims, the review advances a reframing of intuition as a legitimate, cognitively integrative form of strategic reasoning—one that bridges somatic awareness, ethical reflexivity, and relational intelligence. This approach directly challenges the rational-emotional binary still dominant in classical economics and psychology (Kahneman, 2011; Simon, 1955), aligning instead with contemporary developments in feminist economics and neuroconstructivist theory (Crenshaw, 1991; Folbre, 2001; Joel and Vikhanski, 2019; Nelson, 2011). Methodologically, it contributes to the literature by triangulating thematic synthesis, bibliometric techniques, and rigorous quality appraisal, while foregrounding the imperative of epistemic inclusion and conceptual clarity in the study of intuitive cognition in leadership.

## 2 Theoretical and conceptual background

Intuition has historically occupied an ambivalent position in decision science, which is celebrated as the hallmark of expertise yet often discredited as emotional or irrational (Kumar and Taneja, 2022). Classical economic models, grounded in rational choice theory, privileged optimization and calculative logic, excluding intuitive processes as unreliable or epistemically inferior (Luoma and Martela, 2021). However, advances in cognitive psychology, behavioral economics, neuroleadership, and feminist theory have reconceptualized intuition as a fast, embodied, and affect-laden form of cognition that complements, rather than contradicts, deliberation (Adinolfi and Loia, 2022; Brătianu and Stăneiu, 2024).

This paradigm shift supports the emergence of a more pluralistic epistemology of leadership and decision-making. Dual-process scholarship exemplifies this transformation, with the System 1/System 2 labels popularized by Kahneman (2011) while building on earlier dual-process distinctions (Evans and Stanovich, 2013). Yet earlier and more integrative contributions—such as Epstein’s (1998) cognitive-experiential self-theory and Dane and Pratt’s (2007) model of intuitive expertise—highlight how intuition is experientially informed, somatically grounded, and socially modulated. Within management scholarship, edited volumes by Marta Sinclair portray intuition as practice-relevant, context-sensitive, and open to calibration in organizational life (Sinclair, 2011b, 2014, 2020), while Amanda Sinclair emphasizes the morally attuned and relational character of intuitive judgment in leadership settings (Sinclair, 2007; Sinclair, 2014).

In this review, we use System 1 and System 2 as functional metaphors rather than modular brain structures. By System 1 we refer to fast, associative, and context-sensitive processes; by System 2 we refer to slower, sequential, and more readily verbalized operations. These families of processes are complementary, and their performance depends on the learning environment and the availability of feedback (Evans and Stanovich, 2013; Hogarth, 2001; Kahneman and Klein, 2009; Klein, 1998; Schneider and Shiffrin, 1977).

Isenman (2020, 2018), contributes a neurobiological and philosophical account in which feelings bridge body and mind, supporting intuitive appraisal and enabling ethical attunement and contextual foresight. Yet, despite such advances, many cognitive models continue to assume universality, overlooking how gender, socialization, and institutional norms shape the development and expression of intuitive capacities. Eagly and Carli (2007) emphasize that leadership remains encoded in masculinized ideals that prioritize analytic control and abstract reasoning.

While many intuition scholars have implicitly framed the construct as gender-neutral or have downplayed its gendered connotations, Gerd Gigerenzer (2023) offers a compelling counterpoint. In The Intelligence of Intuition, he argues that one of the reasons intuition has historically been marginalized in organizational and cognitive science is precisely because it has been perceived as inherently feminine, in contrast to the rational, masculinized ideals privileged in modern science and management. This tension—between the feminization of intuition and its epistemic devaluation—directly supports the central thesis of this review and adds further historical depth to the structural exclusion of intuitive knowing in economic and leadership discourse.

Complementing Gigerenzer’s sociocultural critique, David Myers (2002) advances the idea that certain types of social intuition may be more readily accessible to women due to evolutionary advantages in social bonding, emotional attunement, and caregiving—traits reinforced through both biological predispositions and cultural reinforcement. While this perspective may risk falling into essentialist frames, it opens the door to considering a more integrative view that does not entirely exclude biological factors from the discussion of intuitive capacities.

Feminist critiques from economics, epistemology, and neuroscience reject these assumptions. Scholars such as Fine (2017a), Gil and Vásquez (1996), and Salazar Montoya and Kew (2023) argue that cognition is relational, embodied, and institutionally situated. Within this framework, female intuition is not a subset of general intuition, but a distinct, multidimensional construct that integrates emotional attunement, contextual sensitivity, embodied knowing, and ethical reflexivity (Isenman, 2018; Sinclair, 2011b).

Drawing on the 142 empirical studies included in this review, four core dimensions of female intuition are identified:

1. Emotional Attunement: More than empathy, this refers to the embodied capacity to detect and interpret affective cues within interpersonal and organizational dynamics. Women frequently use this capacity to anchor decisions in relational and moral clarity, extending Goleman’s (1995) model of emotional intelligence (Salazar Montoya and Kew, 2023; Sinclair, 2011b).
2. Contextual Sensitivity: It entails integrating tacit cues and situational dynamics into decision-making. Gendered socialization and leadership expectations may cultivate women’s adaptive awareness of institutional and interpersonal climates, enabling resilience and innovation under uncertainty (Eagly and Carli, 2007; Hallo and Nguyen, 2021; Khushk et al., 2022).
3. Embodied Cognition: Anchored in interoception and affective coherence, this dimension highlights how somatic awareness informs ethical and strategic judgments. Empirical studies suggest enhanced emotion–cognition integration in women—such as limbic–prefrontal coupling—which supports real-time ethical navigation (Aithal and Satpathy, 2024; Fine, 2017a; Isenman, 2018).
4. Ethical Reflexivity: Prioritizes moral presence, relational accountability, and care, often preceding rational analysis. It frequently manifests as somatic dissonance or intuitive discomfort in the face of ethical complexity (Delaney et al., 2014; Sinclair, 2007).

These four dimensions collectively reframe female intuition as a valid and strategic form of reasoning—one rooted in embodied experience, relational knowledge, and moral discernment. This perspective aligns with foundational critiques in feminist economics and moral philosophy. Scholars like Nelson (2011) and Folbre (2001) challenge abstraction-centered models, advocating instead for epistemologies of care anchored in responsibility and relational agency. Sen’s (2009) capabilities approach complements this view, supporting substantive rationality embedded in agency and ethical concern.

Importantly, the recognition and legitimacy of female intuition vary widely across cultural and institutional contexts. In collectivist societies such as those in Latin America or East Asia, intuitive forms of reasoning are often valued; by contrast, in hyper-rational Western institutions, they remain marginalized—particularly when expressed by women (Gil and Vásquez, 1996; Hallo and Nguyen, 2021; De Sousa Santos, 2014). These disparities expose structural asymmetries in epistemic authority and institutional validation.

This review does not invoke female intuition as an essentialist category, but as an analytical construct aimed at restoring conceptual legitimacy to a historically excluded domain. Grounded in a neuroconstructivist and intersectional framework, the analysis challenges both biological determinism and epistemic universalism (Crenshaw, 1991; Fine, 2017a). It defines female intuition as a cognitively integrative, ethically responsive, and contextually embedded form of strategic reasoning—modulated by social identity, lived experience, and institutional dynamics (Aithal and Satpathy, 2024; Ospina and Foldy, 2009).

This conceptual framework underpins the empirical and theoretical synthesis that follows.

## 3 Methodology

This review was conducted in accordance with the Preferred Reporting Items for Systematic Reviews and Meta-Analyses (PRISMA 2020) guidelines (Page et al., 2021). The protocol was preregistered with the Open Science Framework (OSF) (Tobías, 2025).

To ensure both conceptual and empirical robustness, the analysis integrates:

1. foundational literature in decision science and feminist theory, and
2. a systematically screened corpus of 142 studies across disciplines and regions.

### 3.1 Research questions and inclusion criteria

The review was guided by four interrelated questions:

1. How is female intuition conceptually defined and distinguished from generalized intuitive cognition?
2. What empirical evidence exists regarding its influence on decision quality, innovation, risk calibration, and leadership effectiveness?
3. Which cultural, structural, and neuropsychological factors moderate its enactment and institutional legitimacy?
4. How can these findings provide a refined, context-sensitive theoretical framework?

Inclusion criteria were as follows:

1. empirical or theoretical engagement with intuitive processes in economic or leadership contexts,
2. explicit relevance to gender or female cognition,
3. peer-reviewed publication between 2000 and 2024,
4. theoretical and methodological clarity,
5. publication in English.

Limiting the review to English-language studies ensured bibliographic consistency, but also introduced epistemic constraints, particularly with regard to underrepresented regions (Alatas, 2006; De Sousa Santos, 2014).

### 3.2 Search strategy and corpus construction

A systematic search was conducted across five academic databases—Scopus, Web of Science, PubMed, PsycINFO, and EconLit—using Boolean operators and controlled vocabulary (e.g., MeSH terms, APA Thesaurus descriptors).

Search strings combined key terms such as intuition, female leadership, gender, decision-making, emotional intelligence, and embodied cognition.

The initial query (February 3–5, 2025) yielded 1,750 records. After removing duplicates, 1,340 studies were independently screened by two reviewers (Cohen’s *κ* = 0.82, 95% CI [0.78–0.86]); discrepancies were resolved by discussion. Based on relevance, empirical grounding, and conceptual clarity, 890 studies were excluded.

The remaining 450 full-text articles were assessed using three validated instruments: the Critical Appraisal Skills Program (2018) for qualitative studies, the Mixed Methods Appraisal Tool (MMAT, 2018) for mixed-methods designs (Hong et al., 2018), and the AMSTAR 2 checklist for systematic reviews and meta-analyses (Shea et al., 2017). Each tool was applied in accordance with its original protocol, focusing on the evaluation of construct clarity, methodological rigor, and ethical transparency. As a result of this appraisal, 300 studies were excluded due to insufficient methodological quality or limited engagement with the construct of intuition.

The final corpus comprises 142 peer-reviewed articles: 120 that met all predefined quality thresholds, and an additional 22 identified through snowball sampling, backward citation tracking, and expert consultation. The complete search protocol—including exact search strings, inclusion and exclusion criteria, and database-specific yields—is detailed in Supplementary File S1 and in the OSF registration record (Tobías, 2025). This open-access repository ensures full compliance with PRISMA 2020 standards for methodological transparency and replicability.

### 3.3 Data extraction and analysis

Metadata were extracted using a structured coding template and compiled into a comparative matrix. This enabled cross-study analysis and triangulation across theoretical and methodological axes (Hong et al., 2018).

A three-tiered synthesis strategy was employed:

1. Narrative synthesis to trace conceptual developments and construct integrative interpretations, following best practices for coherence and theoretical contextualization in systematic reviews (Biesta, 2010).
2. Thematic analysis to identify core patterns and emergent domains.
3. Bibliometric mapping using VOSviewer to visualize citation clusters, epistemic influence, and co-occurrence networks (Boell and Cecez-Kecmanovic, 2015; van Eck and Waltman, 2010).

This triangulated design is represented in Supplementary Figure S1, which illustrates the integrative logic linking narrative synthesis, thematic coding, and bibliometric analysis. Full coding procedures, cluster composition, and visualization outputs are detailed in Supplementary Tables S1–S3, hosted via the OSF repository.

In line with integrative review conventions, not all 142 studies are cited within the body of the manuscript. Rather, selected representative works are discussed to illustrate critical findings across the five thematic domains.

### 3.4 Study selection and PRISMA 2020 flow diagram

**Figure 1:**
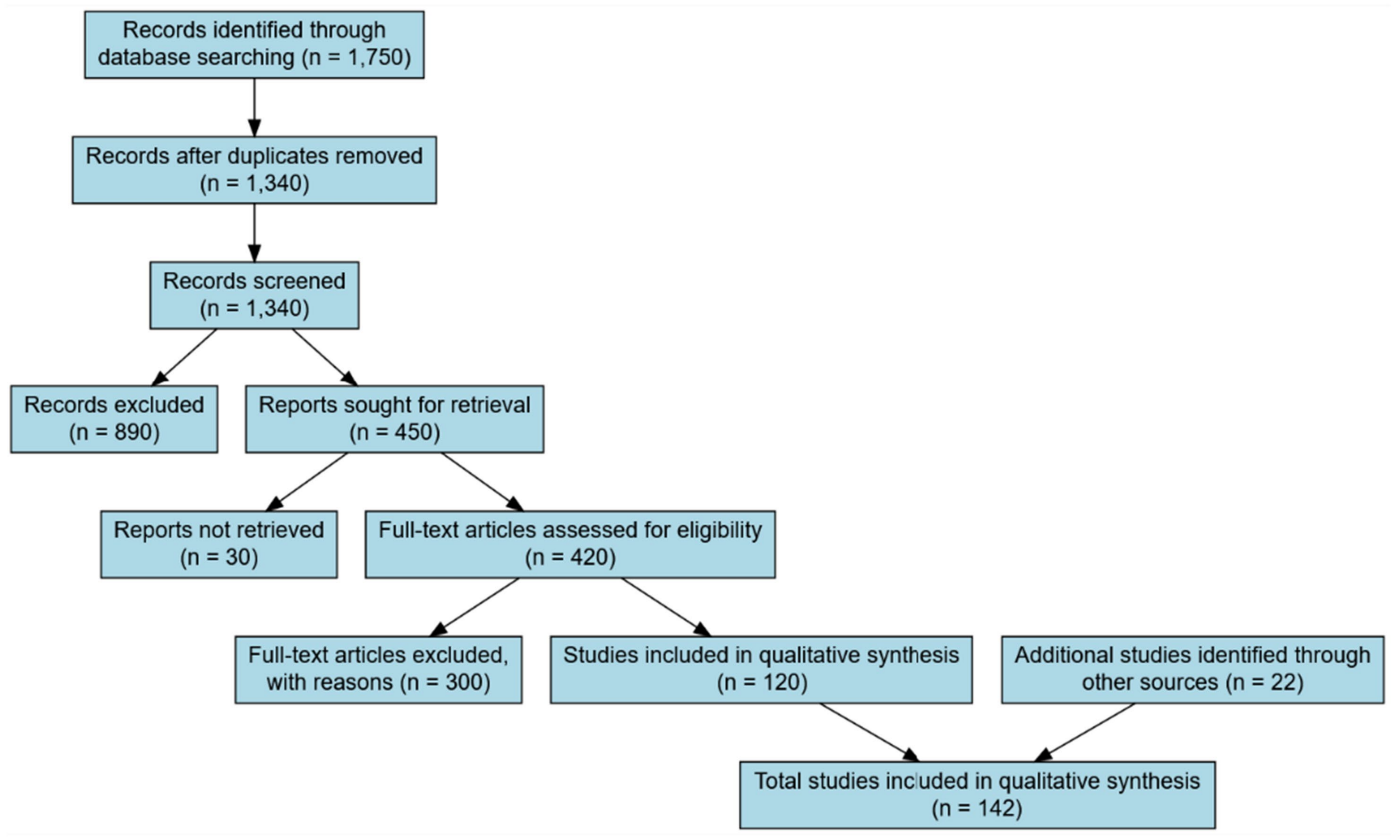
PRISMA 2020 flow diagram of study selection process.

The screening and selection process is summarized in Figure 1 (PRISMA 2020 Flow Diagram). All phases—from database querying to full-text review—were conducted between early and mid-February 2025, following PRISMA 2020 standards (Page et al., 2021).

Title and abstract screening was independently performed by two reviewers, yielding high inter-rater reliability (Cohen’s *κ* = 0.82), consistent with benchmarks for categorical agreement (Cohen, 1960). Discrepancies were resolved through consensus.

To exemplify the quality appraisal process, five studies—each representing a primary design (qualitative, quantitative, or mixed methods)—were evaluated in detail using CASP, MMAT (2018), and AMSTAR 2. The results are shown in Table 1, which demonstrates variation in construct clarity, theoretical alignment, methodological transparency, and risk of bias.

**Table 1:** Quality appraisal of five representative studies.

Study (Author, Year)	Design/Method	Appraisal Tool (Version)	Screening ✓	Critical Items Met n/N (%)	Non-critical items met n/N (%)	Risk of Bias†	Overall Confidence/Rating‡	Included in synthesis
Brunetto et al. (2023)	Mixed Methods (Explanatory)	MMAT 2018	Yes	5/5 (100%)	—	Low	**** (Very High)	Yes
Sitorus and Lusianah (2023)	Qualitative (Grounded Theory)	CASP 2018	—	8/10 (80%)	—	Moderate	Good	Yes
Lauriola et al. (2014)	Systematic Review	AMSTAR 2	Yes	7/7 (100%)	8/9 (89%)	Low	High	Yes
Dunkley (2024)	Narrative Review	CASP 2018	—	7/10 (70%)	—	Moderate	Moderate	Yes
Thrasher et al. (2023)	Qualitative (Narrative Inquiry)	CASP 2018	—	9/10 (90%)	—	Low	Good	Yes

✓ Screening = Study meets both filter questions (relevance and appropriate design).

†Risk of Bias is derived from the algorithms of each appraisal tool:

➤ MMAT 2018: “Low” if ≥4 domain-specific items are met.

➤ AMSTAR 2: “Low” if no critical items are missing.

➤ CASP: “Low” = few limitations; “Moderate” = some limitations.

‡Overall Confidence/Rating:

➤ MMAT: 1–4 stars (e.g., **** = Very High Confidence).

➤ AMSTAR 2: Native scale (High/Moderate/Low/Critically Low).

➤ CASP: Adapted to Good/Moderate/Poor for consistency across tools.

Critical Items.

➤ MMAT 2018 includes 5 domain-level items considered critical.

➤ AMSTAR 2 designates 7 critical domains: Items 2, 4, 7, 9, 11, 13, 15.

➤ CASP does not distinguish; all 10 items are treated as critical here for consistency.

— = Not applicable. MMAT and AMSTAR 2 do not specify non-critical items; CASP weighs all items equally.

The full appraisal matrix covering all 142 studies is presented in Supplementary Table S1 and is available via the OSF registration (Tobías, 2025), enabling independent verification and full methodological traceability.

To reinforce analytical transparency and link evidence to the research aims, Table 2 maps the four research questions (RQ1–RQ4) to the corresponding thematic domains. This matrix ensures that the synthesis is not only methodologically sound but analytically responsive.

**Table 2:** Mapping research questions to thematic domain.

Research question	Addressed in domain(s)	Analytical section(s)	Supporting figures/tables
RQ1 – Conceptual clarity	Domain 1: Conceptualization	Section 4.1	Table 3; Figure 5
RQ2 – Impact on decision-making and innovation	Domains 2–4	Sections 4.2–4.4	Table 4; Figures 6, 7
RQ3 – Moderating factors	Domain 5	Section 4.5	Table 5; Figure 8
RQ4 – Theoretical integration	All domains	Sections 5.1–5.4	Table 5; Figures 7, 8

### 3.5 Bibliometric visualization

To complement the qualitative synthesis, a bibliometric analysis of the 142-study corpus was conducted using VOSviewer (van Eck and Waltman, 2010). This technique enables the detection of semantic proximities, thematic clusters, and conceptual evolution over time, aligning with established science mapping protocols (Boell and Cecez-Kecmanovic, 2015). For a step-by-step depiction of the analytical process, see Supplementary Figure S2.

**Figure 2:**
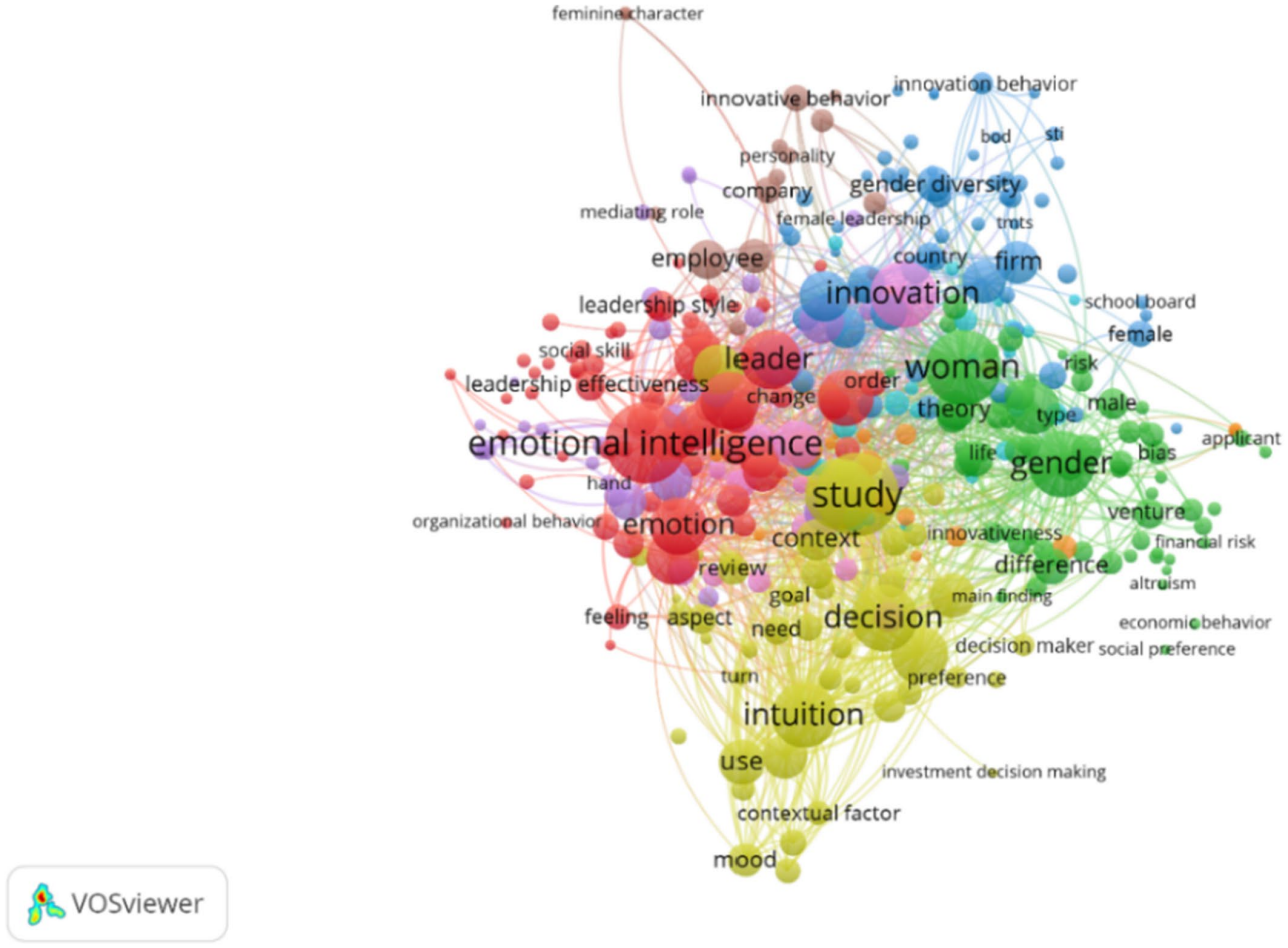
Conceptual network map of keywords in studies on female intuition (2000–2024).

Figure 2 presents the keyword co-occurrence network derived from terms appearing at least five times across the dataset. Five thematic clusters emerged through LinLog modularity optimization, visually distinguishing areas of conceptual density related to emotional intelligence, gendered innovation, intuitive decision-making, contextual cognition, and neurocognitive diversity. These clusters directly mirror the five analytical domains elaborated in the qualitative synthesis.

**Figure 3:**
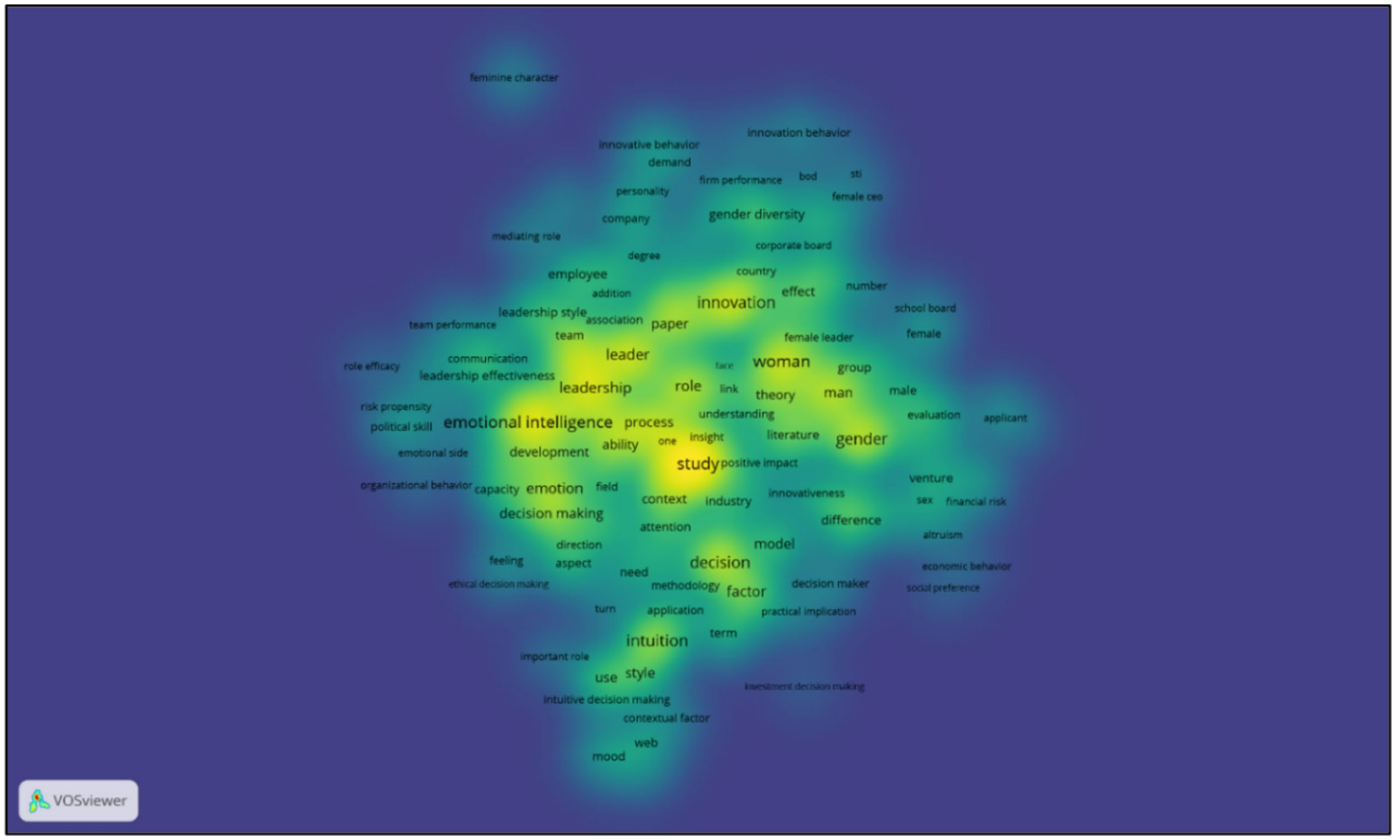
Keyword density map highlighting cognitive and relational constructs (2000–2024).

Building on this, Figure 3 displays a keyword density map constructed through Gaussian kernel estimation. This visualization reveals areas of epistemic saturation—terms such as ethical reflexivity, embodied cognition, and relational leadership appear as focal points of cumulative scholarly attention. Warmer zones indicate a higher concentration of conceptual relevance, offering a proxy for thematic centrality across the corpus.

**Figure 4:**
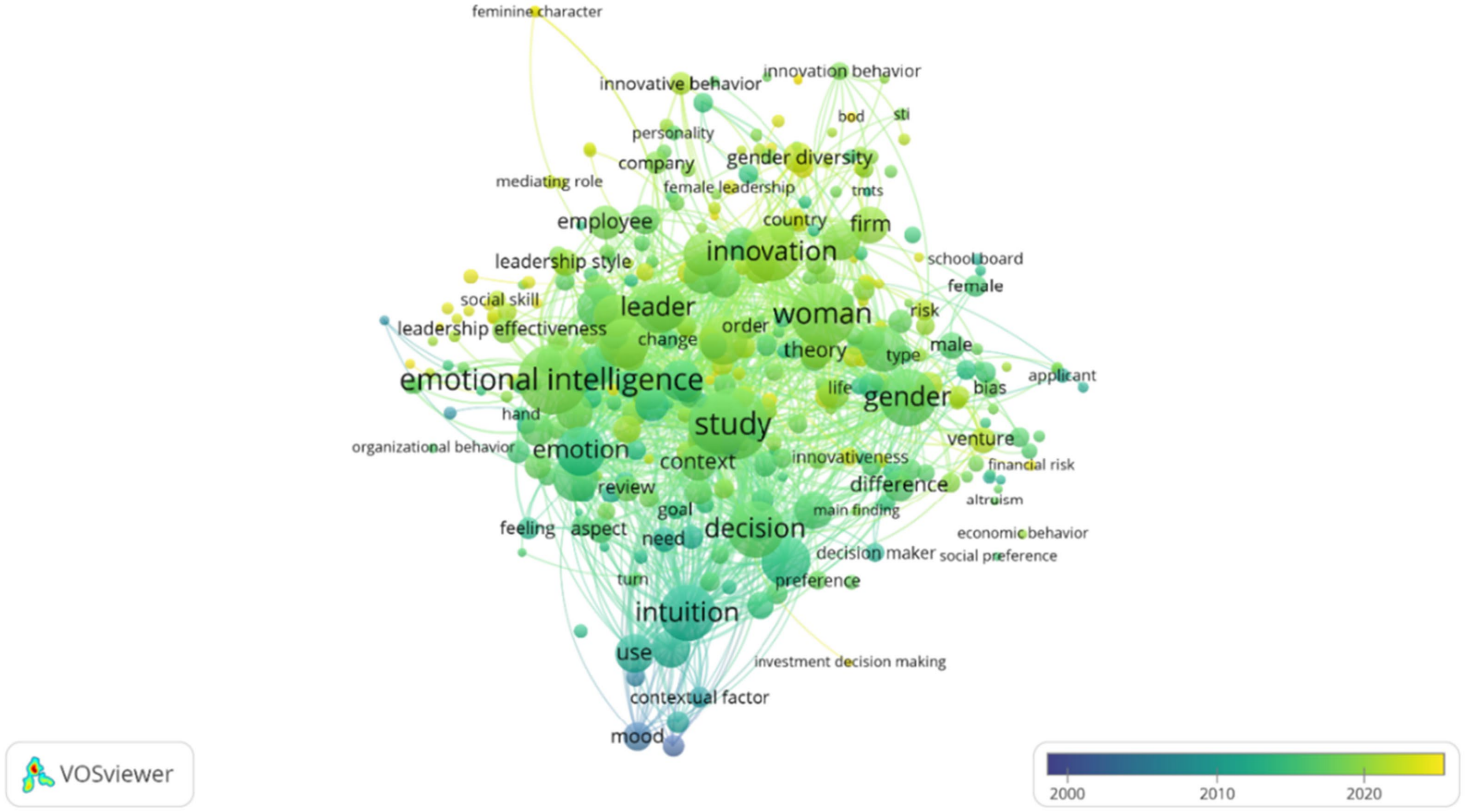
Temporal overlay of keyword emergence in female intuition research (2000–2024).

Figure 4 offers a temporal overlay illustrating the emergence of keywords from 2000 to 2024. Each term is positioned along a color-coded gradient representing its average year of occurrence. This temporal visualization reveals an intensification of interest in constructs such as intuitive ethics, intersectionality, and embodied leadership—concepts that have gained empirical and theoretical traction particularly in the past decade.

Taken together, these bibliometric visualizations both validate and enrich the thematic architecture constructed through qualitative synthesis. They also uncover emergent conceptual nodes—such as the alignment between embodied cognition and ethical leadership—that remain peripheral in mainstream cognitive and economic models of decision-making (Lauriola et al., 2014; Sadler-Smith, 2008), yet appear central to a multidimensional reconceptualization of female intuition.

### 3.6 Integrative bridge: answering the research questions

The methodological synthesis established above forms a coherent scaffold for addressing the guiding questions:

RQ1: Definition and Distinction: Addressed by Domain 1, which operationalizes female intuition as a four-dimensional construct: emotional attunement, contextual sensitivity, embodied cognition, and ethical reflexivity.

RQ2: Influence on Decision and Innovation: Domains 2, 3, and 4 demonstrate how female intuition recalibrates risk, enhances leadership, and fosters innovation in high-stakes contexts.

RQ3: Moderating Forces: Domain 5 identifies cultural, structural, and neuropsychological variables that modulate the expression and legitimacy of female intuition, moving beyond essentialism.

RQ4: Toward a Framework: Insights converge in Section 5, where a neuroconstructivist and intersectional model of female intuition is proposed as a cognitively integrative, ethically grounded, and strategically situated resource.

Together, these mappings ensure that the synthesis is both thematically coherent and analytically responsive—clarifying what female intuition is, how it operates, when it is effective, and why it merits recognition.

## 4 Results

Synthesizing evidence from 142 peer-reviewed studies, this review describes intuitive cognition—observable in leaders of all genders but especially well-documented among women—as a form of anticipatory intelligence that:

- recalibrates risk perception under uncertainty,
- amplifies transformational leadership through ethical and relational attunement,
- in both female and male leaders can foster innovation by aligning strategy with stakeholder complexity, and
- is legitimized to different degrees across cultural, institutional, and neurocognitive contexts.

Although not exclusive to women, the evidence shows that female leaders mobilize these capacities through stronger relational scanning and ethical framing. The five domains below elaborate these contributions and their relevance to contemporary leadership theory and practice.

### 4.1 Conceptualization of female intuition

Contemporary literature increasingly frames intuition as a dual-process cognitive mechanism—one that complements analytical reasoning by enabling rapid, affect-rich judgments under uncertainty (Hallo and Nguyen, 2021; Krava et al., 2021). Within this framework, female intuition is not merely a stylistic variant but emerges as a distinct, relationally embedded modality shaped by sociocultural norms (Eagly and Carli, 2007; Gil and Vásquez, 1996), neurocognitive dynamics (Aithal and Satpathy, 2024; Isenman, 2018), and leadership experiences situated within gendered institutional environments (Delaney et al., 2014; Sinclair, 2011a). Women in executive and entrepreneurial contexts often activate this form of reasoning, characterized by emotional attunement, contextual acuity, and embodied sensemaking (Downey et al., 2006; Song, 2024). See Supplementary Figure S3 for a visual synthesis of these converging neuro- and sociocultural pathways.

Recent neuroscientific research has deepened our understanding of the mechanisms underpinning gendered intuitive cognition. Studies by Hallo and Nguyen (2021) and Aithal and Satpathy (2024) document sex-related differences in affective neural activation and limbic–prefrontal integration, which support adaptive decision-making in emotionally complex scenarios. These findings align with broader evidence from neuroleadership and affective neuroscience, suggesting that women tend to exhibit greater cross-hemispheric connectivity and emotional modulation under conditions of uncertainty (Gur and Gur, 2017; Zak, 2012). When emotional intelligence is high, women frequently combine intuitive recognition with analytical structuring, enhancing both relational insight and ethical foresight (Downey et al., 2006; Isenman, 2018).

Cultural narratives further influence the legitimacy of intuitive cognition. As Salazar Montoya and Kew (2023) observe, societal expectations around empathy and reflexivity can institutionalize intuitive expertise in women—although the degree of legitimation varies by context. In Latin America, such cognitive styles may be affirmed or constrained by prevailing institutional logics (Guillén and Pereira, 2022). Within feminist economics and leadership studies, Amanda Sinclair argues that embodied, context-sensitive modalities constitute legitimate and strategic forms of situated knowing in practice (Sinclair, 2007, 2011a).

Empirical research confirms that, when integrated with emotional intelligence, female intuition enables leaders to anticipate stakeholder needs, navigate complexity, and resolve ethical tensions effectively (Downey et al., 2006; Salazar Montoya and Kew, 2023). In this light, it emerges as a neurocognitively grounded and socially cultivated form of reasoning.

**Figure 5:**
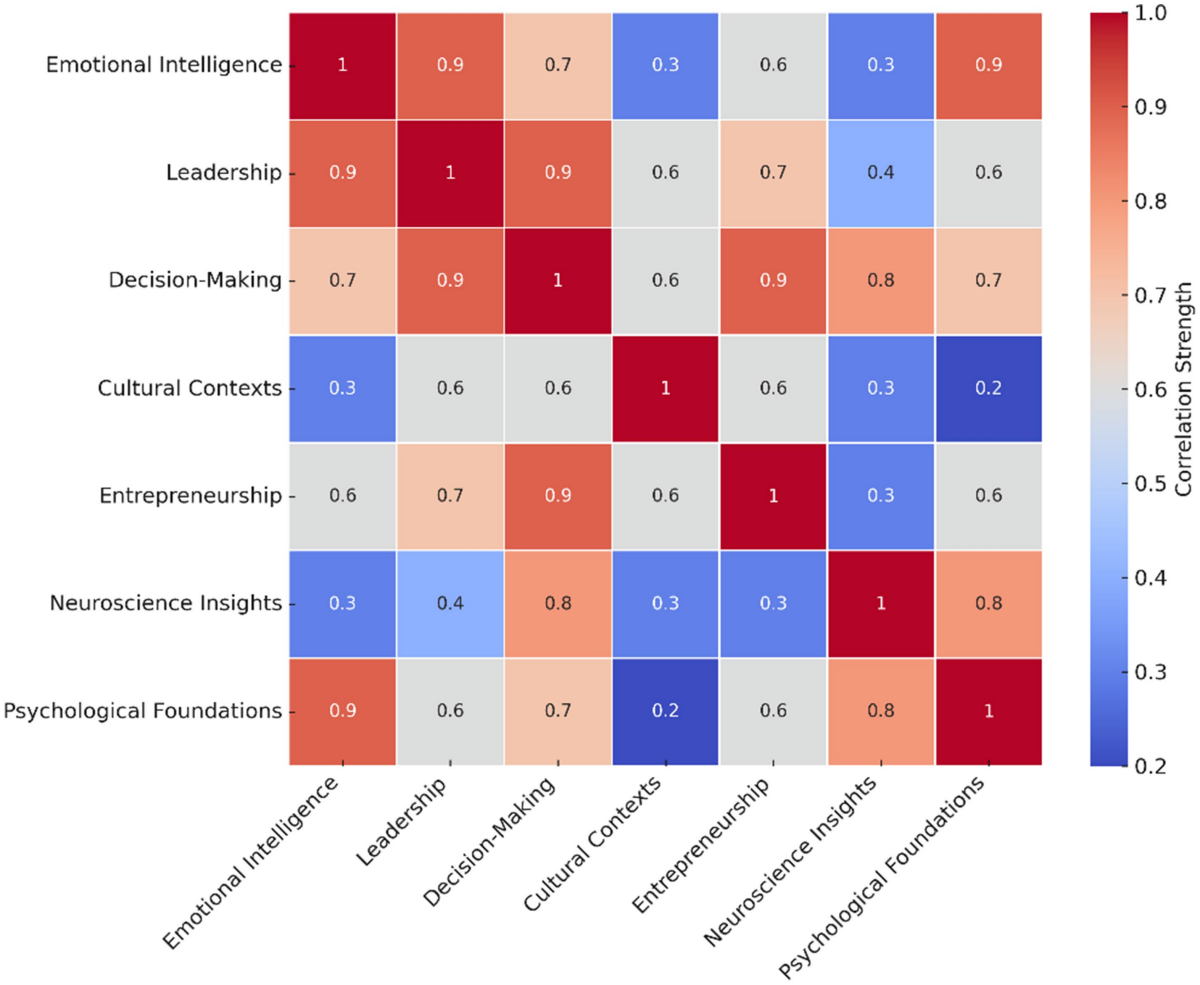
Interdomain correlation heatmap of female intuition constructs (2000–2024).

To visualize the structural coherence of the female intuition construct, Figure 5 presents a correlation heatmap generated from term co-occurrence analysis across seven key domains. This bibliometric mapping approach reveals latent thematic clusters and strong interconnections—particularly among emotional intelligence, leadership, and ethical decision-making. These results empirically support a multidimensional conceptualization of female intuition, in line with established methods in science mapping and conceptual clustering (Boell and Cecez-Kecmanovic, 2015; van Eck and Waltman, 2010).

This figure presents a heatmap of Spearman correlation coefficients across the core constructs of female intuition identified in the systematic review. The use of Spearman’s rho accounts for the non-parametric distribution of the data. Variables include contextual sensitivity, embodied cognition, ethical reflexivity, and emotional attunement. Higher values indicate empirical co-occurrence patterns based on their joint appearance and conceptual proximity across the 142 analyzed studies. These correlations do not imply causation but suggest meaningful associative trends that support the multidimensional architecture of female intuition.

To complement these empirical findings, a theoretical synthesis is presented below to distinguish female intuition from adjacent cognitive constructs. While Figure 5 captures relational patterns within the intuition framework, the following matrix (Table 3) offers a conceptual comparison that clarifies overlaps and divergences with related constructs such as emotional intelligence, general intuition, impulsivity, and affective heuristics.

**Table 3:** Comparative matrix of female intuition and adjacent constructs.

Construct	Core features	Epistemological base	Overlaps with female intuition	Key differences
Female Intuition	Embodied, affective, relational, context-sensitive, ethical	Dual-process theory, feminist epistemology	Emotional intelligence, ethical reflexivity	Situated expertise, gendered cognition, neuro-sociocultural integration
General Intuition	Rapid, automatic, tacit knowledge- based	Dual-process theory	Pattern recognition	Often decontextualized and not gender-sensitive
Emotional Intelligence	Emotional awareness, regulation, empathy	Social and behavioral psychology	Affective attunement, empathy	Lacks embodied/somatic and strategic foresight dimensions
Impulsivity	Fast, affect-driven, low deliberation	Neuropsychology, behavioral economics	Rapid judgment	Lacks ethical, contextual, and reflective depth
Affective Heuristics	Emotion-based shortcuts in judgment	Cognitive psychology, behavioral finance	Emotionally grounded processing	Lacks moral reflexivity and embodied ethical integration

This matrix delineates core features, epistemological bases, areas of convergence, and key differences among constructs often conflated with female intuition. It aims to reduce conceptual slippage—particularly between intuitive affect and emotionally grounded shortcuts—by highlighting the situated, ethical, and somatic dimensions of female intuitive reasoning (Damasio, 2021; Sadler-Smith, 2008; Hogarth, 2001).

Recent empirical studies further substantiate these distinctions. Brunetto et al. (2023) and Imran et al. (2023) demonstrate that emotional intelligence mediates the relationship between female leadership and innovative decision-making. Likewise, Sumanth et al. (2023) show that intuitive leadership grounded in psychological insight enhances team adaptability and trust. Collectively, these findings support the proposition that female intuition is not a static trait, but a neurocognitive and relational composite—dynamically shaped by context, emotional fluency, and embodied awareness (Bacha and Niesten, 2024).

### 4.2 Risk perception and decision-making

Female intuition plays a pivotal role in reshaping how risk is perceived and managed, directly challenging longstanding stereotypes of gendered risk aversion (Croson and Gneezy, 2009; Eagly and Carli, 2007). Rather than reflecting impulsivity, intuitive reasoning in women leaders often embodies ethically attuned and context-sensitive cognition—a mode of awareness that enables proactive alignment with stakeholder needs in conditions of uncertainty (Delaney et al., 2014; Downey et al., 2006). This perspective reframes risk-taking not as a deviance from rational control, but as a relationally embedded capacity shaped by emotional intelligence and environmental responsiveness.

Empirical evidence from finance and entrepreneurship substantiates this view. Peni (2014) found that female-led firms exhibit higher levels of innovation-related risk-taking. Downey et al. (2006) and Klenke (2005) observed that women in senior executive roles frequently engage affective-intuitive frameworks when navigating volatility, particularly in the aftermath of organizational crises. Fellnhofer (2022) notes that intuition can be consciously cultivated as a strategic capability that enhances foresight and adaptive agility.

Social context significantly modulates how intuitive cognition is enacted and legitimized. Sekścińska et al. (2023) show that activating gendered social roles alters risk preferences, while Delaney et al. (2014) find that female entrepreneurs often integrate tacit, scenario-based reasoning into their uncertainty management practices. In collectivist societies such as Nigeria and the Philippines, intuition is institutionally validated as an accepted component of leadership (Kolade et al., 2020). By contrast, in Latin American contexts, structural asymmetries and prevailing institutional logics frequently limit its epistemic visibility (Guillén and Pereira, 2022). In data-driven and rationalist organizational cultures, intuitive insights may require translation into analytical formats to gain recognition (Eling et al., 2015; Hallo and Nguyen, 2021).

Taken together, this body of literature suggests that female intuition operates as a form of anticipatory intelligence—ethically responsive and relationally grounded. When supported by institutional cultures and inclusive leadership norms, it facilitates more holistic decision-making, especially under conditions of volatility and ambiguity (Ingersoll et al., 2023).

These empirical insights are synthesized in Table 4, which maps how female intuition influences risk perception across diverse cultural and organizational environments. The table highlights key mechanisms—such as emotional regulation, stakeholder attunement, and tacit foresight—through which intuitive cognition allows women leaders to engage with uncertainty not as a threat, but as a relational and ethically navigable challenge.

**Table 4:** Female intuition and risk perception: empirical evidence across contexts.

Study	Context	Method	Key findings
Peni (2014)	Publicly traded firms (Europe)	Quantitative (Panel Data)	Female CEOs demonstrate strategic initiative and higher innovation intensity in complex environments, challenging gendered risk aversion assumptions.
Klenke (2005)	Post-9/11 Organizational Strategy	Qualitative (Case Study)	Women leaders used morally anchored intuition in crisis, enabling rapid, ethically responsive navigation of existential uncertainty.
Sekścińska et al. (2023)	Behavioral Finance Experiment	Experimental Psychology	Activating gender-related social roles influenced financial risk-taking; feminine roles reduced it, showing how intuitive responses are socially modulated.
Delaney et al. (2014)	Female Entrepreneurs (Australia)	Qualitative (Narrative Interviews)	Intuitive foresight enabled scenario-based planning in high-volatility startup contexts.
Kolade et al. (2020)	Entrepreneurial Firms (Nigeria)	Mixed Methods	Intuition legitimized by collectivist values; anticipatory strategies enhanced resilience and stakeholder trust.
Ingersoll et al. (2023)	Female CEOs (U. S. Firms)	Quantitative (Panel Data)	Women using context-sensitive and intuitive reasoning achieved more stable firm performance under volatile market conditions.
Downey et al. (2006)	Senior Female Managers	Quantitative (Psychometric Assessment)	The combination of intuition and emotional intelligence in female leaders enhances ethical decision-making and effectiveness in socially complex organizational settings.

These findings resonate with integrative models of intuitive cognition. Rather than reducing female intuition to affective automaticity, frameworks such as Epstein’s (1998, 2010) experiential system and Dane and Pratt’s (2007) intuitive expertise model reveal how tacit pattern recognition, emotional attunement, and somatic markers inform adaptive risk navigation. When combined with Goleman’s (1995) emotional intelligence theory and Eagly and Carli’s (2007) work on leadership legitimacy, these frameworks position female intuition as a core dimension of relational risk governance—contextually anchored and morally responsive.

Beyond individual cognitive traits, this capacity supports broader paradigms of ethical and transformational leadership. The ability to anticipate, recalibrate, and ethically navigate risk through intuitive reasoning positions female intuition as a vital resource in volatile, complex organizational landscapes—particularly when analytical models fall short in ambiguous contexts (Bass and Riggio, 2006; Sinclair, 2011b). Empirical studies confirm that intuition, when integrated with emotional and ethical intelligence, enables rapid, morally attuned responses attuned to both stakeholder dynamics and institutional values (Aithal and Satpathy, 2024; Goleman, 1995).

This foundation establishes a strong empirical and theoretical bridge toward the next domain, where female intuition functions not only as a mechanism for decision calibration, but as a key enabler of transformational influence and relational leadership.

### 4.3 Transformational leadership and organizational impact

A growing body of empirical and theoretical evidence links female intuition to transformational leadership—a leadership paradigm characterized by vision, empathy, moral grounding, and adaptive influence (Bass and Riggio, 2006). Unlike transactional leadership, which emphasizes control and exchange, the transformational model fosters trust, creativity, and psychological investment—dimensions in which women often excel, due in part to elevated emotional intelligence and intuitive attunement (Downey et al., 2006; Ghouse, 2023).

Cross-sectoral studies confirm the consistency of this dynamic. During the COVID-19 crisis, female heads of state and institutional leaders demonstrated relational and ethically attuned responses that stabilized public trust (De Blasio and Selva, 2020; UN Women, 2021). In education systems affected by conflict, women deployed intuitive strategies to maintain institutional cohesion and psychological resilience (Muzayanah and Anggraeni, 2023; Ruggs et al., 2023). Likewise, entrepreneurial leaders draw on affective foresight and stakeholder attunement to navigate complexity and innovation (Ghouse, 2023; Kolade et al., 2020).

Neuroscientific insights further substantiate these patterns. Research indicates that women demonstrate greater interhemispheric integration and oxytocin-mediated responsiveness, facilitating intuitive synthesis and trust calibration under pressure (Aithal and Satpathy, 2024; Hurlemann and Scheele, 2016). These neurobiological processes support the rapid moral alignment and interpersonal resonance that are essential to transformational leadership. Zhu et al. (2022) and Song (2024) additionally report that intuitive cognition enables early detection of relational tension and fosters responsive leadership adjustments in high-stakes settings.

Importantly, this capacity cannot be reduced either to biological determinism or to sociocultural scripts taken in isolation. Current evidence points to a reciprocal dynamic in which neurobiological plasticity is sculpted by gendered experience, and vice-versa (Fine, 2017a; Gur and Gur, 2017; Joel and Vikhanski, 2019). Through socialization, women often cultivate advanced capacities for empathy, relational communication, and moral responsiveness, which—when combined with the somatic-marker and inter-hemispheric mechanisms detailed in Section 5.4—enhance their ability to govern intuitively in institutional settings (Eagly et al., 2003). In sectors such as fintech and social enterprise, this translates into tangible strategic value: women leaders blend foresight and cultural agility to manage innovation while fostering psychological safety (Brunetto et al., 2023; Imran et al., 2023).

Crucially, intuition complements—not replaces—analytical reasoning. As Hodgkinson and Sadler-Smith (2017) emphasize, effective decision-making under uncertainty depends on the dynamic interplay between intuitive and deliberative processes. In volatile markets, overreliance on metrics may obscure emerging needs. Leaders who successfully integrate intuitive discernment with analytical modeling demonstrate superior agility and ethical foresight (Hallo and Nguyen, 2021; Ingersoll et al., 2023).

Drawing on Miller and Ireland’s (2005) taxonomy, we distinguish automated expertise (AE)—rapid pattern recognition grounded in extensive domain rehearsal—from the holistic hunch (HH), a cross-domain, affect-laden synthesis that surfaces under novel, weakly structured conditions (Dane and Pratt, 2007; Sadler-Smith, 2008). AE yields fast, high-fidelity judgments when cue validity is known and feedback cycles are tight. HH integrates somatic markers (Damasio, 2021), contextual sense-making, and moral imagination to generate anticipatory guidance precisely when statistical regularities are sparse or ambiguous. The two modes are complementary: AE contributes fine-grained micro-signals; HH supplies integrative macro-framing—together enabling leaders to reconcile local accuracy with systemic relevance. Emerging neurobiological evidence of sex-linked patterns in functional connectivity hints that female leaders may shift more fluidly between AE and HH as uncertainty rises (Hurlemann and Scheele, 2016).

**Figure 6:**
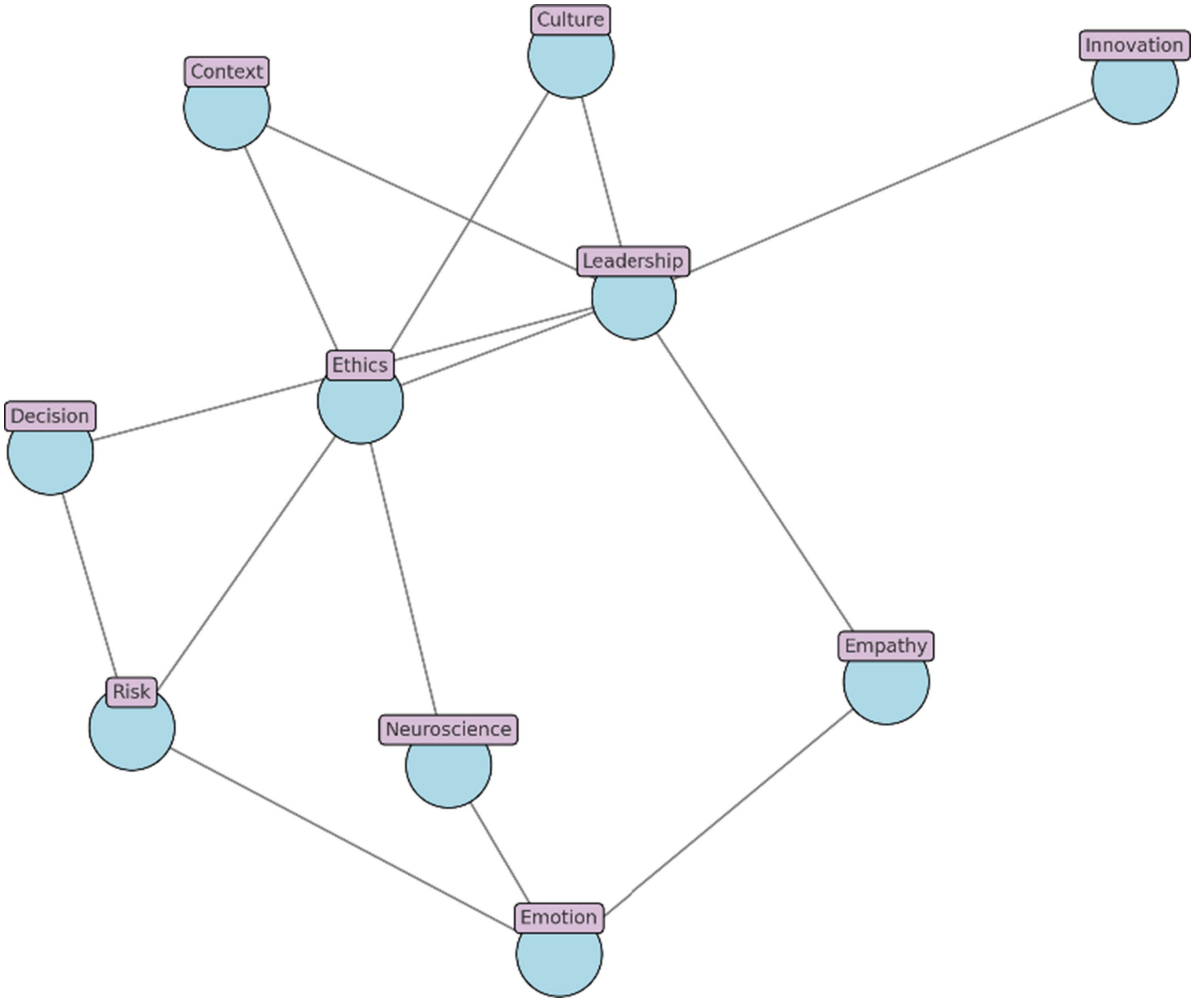
Intuitive leadership as a relational-cognitive network.

This AE–HH complementarity underpins Figure 6, which models intuitive leadership as an integrated network drawing on Goleman’s (1995) emotional intelligence, Sinclair’s (2011a) affective intuition as relational compass, and Eagly and Carli’s (2007) gendered legitimacy lens. The model illustrates how intuitive cognition can catalyze ethical responsiveness, affective resonance, and adaptive leadership in volatile, morally complex institutional environments.

Transformational leadership is thus redefined through female intuition. As Eagly and Chin (2010) and Ospina and Foldy (2009) highlight, leadership must increasingly be understood as an embodied, relational, and morally situated process. It constitutes a form of ethical influence grounded in embodied cognition, relational capital, and adaptive responsiveness—dimensions often overlooked by traditional evaluation frameworks. Far from contradicting data-driven governance, intuitive reasoning ensures that human nuance and ethical discernment remain central to complex decision-making (Floridi and Cowls, 2021; Khatri and Ng, 2000).

In the digital era—where algorithmic logic risks displacing moral complexity—female intuition offers a corrective epistemic architecture. As argued by Goleman (1995) and Fine (2017a), emotional and contextual intelligence function as critical counterweights to systematized reasoning. They enable leaders to bridge technocratic systems with situated discernment, integrating emotion, context, and moral reasoning (Bas et al., 2022; Sadler-Smith and Héliot, 2021; Isenman, 2020). In this light, embodied cognition resists epistemic homogenization and affirms the legitimacy of plural, context-sensitive leadership pathways.

### 4.4 Innovation, entrepreneurship, and strategic agility

Female intuition has gained increasing recognition as a strategic cognitive asset in contexts marked by innovation and adaptive decision-making—particularly under conditions of volatility, complexity, and high ambiguity (Ghouse, 2023). Its non-linear, emotionally attuned character enables women to detect emerging opportunities, align stakeholder expectations, and navigate uncertainty with ethical foresight (Bacha and Niesten, 2024; Delaney et al., 2014; Kolade et al., 2020). Leaders who fluidly alternate between automated expertise and holistic hunch translate routine efficiency into exploratory breakthroughs, a combination that recent studies associate with higher innovation yields under radical uncertainty (Gigerenzer, 2023; Hadi et al., 2024).

Empirical studies consistently show that women leaders leverage intuitive foresight, contextual sensitivity, and ethical framing to catalyze innovation—especially in emerging markets and resource-constrained environments (Khushk et al., 2022; Madison et al., 2022). Supplementary Table S3, available via the OSF registration (Tobías, 2025), synthesizes the mechanisms that underlie this cognitive capacity, emphasizing the importance of institutional conditions—such as inclusive cultures and psychological safety—in legitimizing intuitive strategic approaches (Brunetto et al., 2023; Sumanth et al., 2023).

Here, intuition operates as an integrative faculty, fusing emotional intelligence, ethical discernment, and strategic judgment into a cohesive decision-making process (Adinolfi and Loia, 2022; Aithal and Satpathy, 2024;). Organizations that acknowledge and validate this modality enable women to mobilize what Bacha and Niesten (2024) term intuitive capital—a form of cognitive resource that drives both innovation and social impact.

The benefits of this faculty are not confined to the individual level. At the team level, research shows that intuitive leadership fosters trust, creative collaboration, and adaptive cohesion. Downey et al. (2006) and Goleman (1995) demonstrate that women’s intuitive management styles enhance relational bonds and group innovation—conditions aligned with transformational leadership frameworks (Bass and Riggio, 2006; Eagly et al., 2003).

Entrepreneurship offers a vivid illustration of these dynamics. In contexts such as Oman and sub-Saharan Africa, intuitive insight enabled female entrepreneurs to pivot during crises and cultivate stakeholder trust (Ghouse, 2023; Kolade et al., 2020). These case studies exemplify how female intuition—through the convergence of embodied awareness, ethical foresight, and contextual sensitivity—supports strategic agility under high uncertainty. Drawing from interdisciplinary evidence, including leadership psychology and cognitive neuroscience (Delaney et al., 2014; Isenman, 2018), this phenomenon positions intuitive cognition as a core enabler of ethically grounded and adaptive decision-making.

Findings from neuroscientific research lend robust support to this view. Studies link women’s anticipatory cognition to stronger integration of emotion regulation and somatic markers—neurophysiological mechanisms aligned with embodied cognition theories (Fine, 2017a; Hallo and Nguyen, 2021; Lauriola et al., 2014). These biological substrates enhance relational agility and strategic responsiveness, particularly when nurtured by supportive institutional environments.

Cultural context also plays a defining role. In collectivist cultures such as Mexico and Vietnam, intuitive reasoning is socially legitimized and integrated into leadership logics (Acevedo-Duque et al., 2021; Guillén and Pereira, 2022). By contrast, in hyper-rationalist systems, intuitive insights must often be translated into analytical formats to gain institutional acceptance—a process that can dilute their originality and strategic potential (Bas et al., 2022; Hallo and Nguyen, 2021; Isenman and Sinclair, 2025; De Sousa Santos, 2014).

Emerging evidence further identifies neurobiological moderators—such as oxytocin—that support prosocial risk-taking and intuitive trust-building within entrepreneurial ecosystems (Aithal and Satpathy, 2024; Zak, 2012). These mechanisms reinforce relational competence, particularly when culturally and structurally supported.

Yet, the translation of intuitive reasoning into rationalist proxies often incurs significant epistemic costs. In performance-driven systems, women may feel compelled to justify intuitive judgments using data artifacts or analytical simulations—an act that undermines the authenticity and innovative potential of their cognition (De Sousa Santos, 2014; Sinclair, 2011a). These tensions expose epistemological hierarchies that privilege calculative logic over embodied and affective forms of knowing.

Acknowledging and addressing these tensions is essential. Innovation ecosystems committed to cognitive pluralism must adopt inclusive evaluative criteria that legitimize intuition as a context-sensitive, ethically attuned mode of strategic reasoning (Fine, 2017b; Khatri and Ng, 2000; Sadler-Smith, 2008). Far from being peripheral, female intuition proves central to resilience, stakeholder alignment, and ethically grounded innovation (Brunetto et al., 2023; Delaney et al., 2014).

Ultimately, the interplay between institutional norms, cultural validation, and neurobiological potential determines whether intuitive cognition is nurtured as a strategic resource—or suppressed as a deviation from rational orthodoxy (Hodgkinson and Sadler-Smith, 2017; Isenman, 2009b). The next section examines these moderating forces in greater depth.

### 4.5 Cultural, structural, and neuropsychological moderators

The expression, epistemic legitimacy, and strategic enactment of female intuition are profoundly shaped by sociocultural, institutional, and neurobiological contingencies. As recent empirical and theoretical work demonstrates, its enactment is not the result of essentialist predispositions, but of an interwoven ecology of narratives, structures, and mechanisms that frame its salience and validation (Aithal and Satpathy, 2024; Salazar Montoya and Kew, 2023; De Sousa Santos, 2014).

This multidimensionality unfolds across macro (cultural narratives), meso (institutional logics), and micro (neuropsychological mechanisms) levels—revealing how each tier interacts to modulate the salience and legitimacy of intuitive cognition in female leadership.

Cultural narratives play a foundational role. In collectivist regions such as East Asia, the Nordic countries, and Latin America, female intuition is often legitimized as a source of moral discernment and relational intelligence (De Blasio and Selva, 2020; Eagly and Carli, 2007; Gil and Vásquez, 1996). However, such legitimacy is frequently fragile and contingent on normative frameworks like *marianismo*—a narrative that may celebrate emotional capacities while simultaneously reinforcing expectations of sacrifice, self-denial, or moral infallibility. These ambivalent archetypes position female intuition as both valued and constrained within patriarchal moral logics (Gil and Vásquez, 1996; Salazar Montoya and Kew, 2023).

In hyper-rational or male-dominated institutional logics, intuitive cognition tends to be discounted unless translated into analytically sanctioned formats (Croson and Gneezy, 2009; De Sousa Santos, 2014). Empirical work shows that women often convert intuitive judgments into metric-compatible rationales to secure legitimacy—sometimes at the cost of authenticity and epistemic justice (Delaney et al., 2014; Sinclair, 2007). Addressing such asymmetries requires not only geographic inclusion but also epistemic diversification—expanding what counts as valid knowledge, and the forms in which it may be articulated, within organizational frameworks (Bas et al., 2022; Isenman and Sinclair, 2025; Sinclair, 2011a; Sinclair et al., 2024).

Structural constraints also function as powerful moderators. Bureaucratic systems grounded in formal evaluation and quantitative metrics often marginalize affective and relational cognition (Hodgkinson and Sadler-Smith, 2017). In contrast, entrepreneurial and crisis-driven environments tend to legitimize intuition as an adaptive resource (Delaney et al., 2014; Ghouse, 2023). Female leaders in nonprofit and educational sectors often navigate institutional pressures by aligning intuitive foresight with performance frameworks—a strategic negotiation of legitimacy (Eagly and Carli, 2007; Gring-Pemble et al., 2024).

Neuropsychological moderators add further complexity. Studies suggest that women exhibit greater interhemispheric integration and emotion-regulation capacity—traits potentially modulated by oxytocin, estrogen, and other hormonal variables (Aithal and Satpathy, 2024; Gur and Gur, 2017). These mechanisms support anticipatory cognition and relational attunement. Yet, from a neuroconstructivist perspective (Fine, 2017a; Joel and Vikhanski, 2019), these capacities are not biologically predetermined but rather context-responsive potentials co-constructed through neural plasticity and social learning.

These insights gain further depth when viewed through the lens of dual-process neuroscience. Lieberman’s (2007) distinction between reflexive and reflective systems challenges traditional biases that equate intuition with irrationality. The reflexive system—centered in the ventromedial prefrontal cortex and the amygdala—supports rapid, affect-laden responses grounded in social and emotional cues. The reflective system—associated with the dorsolateral prefrontal cortex—enables deliberative reasoning, abstraction, and self-monitoring. Rather than opposing each other, these systems form a complementary architecture that legitimizes intuitive cognition as a parallel and evolutionarily adaptive mode of reasoning.

Empirical studies suggest that women may exhibit more fluid integration between these systems, facilitating the incorporation of contextual, emotional, and interpersonal information into decision-making (Aithal and Satpathy, 2024; Gur and Gur, 2017). This neurocognitive synergy reinforces the claim that female intuition is not only experientially grounded but also neurobiologically plausible—offering a scientific counterweight to androcentric frameworks that privilege disembodied rationality.

While we do not adopt an essentialist position, it is important to acknowledge evolutionary and social hypotheses—such as those proposed by Myers (2002)—which suggest that women may have developed a predisposition toward intuitive social reasoning due to caregiving and affiliative roles in early human groups. Though contested, such perspectives contribute to framing intuition as a socially embedded and adaptively meaningful phenomenon. From a neuroconstructivist standpoint, however, these dispositions are understood not as immutable traits, but as dynamic affordances shaped by developmental, cultural, and institutional variables.

Cultural archetypes further mediate interpretive frameworks. In Confucian-influenced societies, intuitive leadership may be congruent with values such as harmony or moral equilibrium yet still subordinated to hierarchical norms and patriarchal structures (Heilemann and Faix, 2023). In Western technocratic systems, data-centric rationality often dominates, overshadowing embodied ways of knowing unless explicitly challenged by inclusive epistemologies (Croson and Gneezy, 2009).

Taken together, these findings affirm that female intuition is a situated form of cognition—not a gendered essence, but a dynamic capacity shaped by social identity, neurobiological plasticity, and institutional architectures (Fine, 2017b; Joel and Vikhanski, 2019; Sinclair, 2011b). Its legitimacy is contingent upon how organizations define knowledge, evaluate decision-making, and distribute epistemic authority (Ospina and Foldy, 2009; Sadler-Smith and Héliot, 2021).

Crucially, this review explicitly rejects essentialist interpretations of female intuition. Neurobiological findings are framed as dispositional affordances rather than fixed traits. Overreliance on hormonal explanations risks reinforcing gender binaries and neglecting the sociocultural scaffolding through which intuition is shaped and expressed. Instead, we adopt a neuroconstructivist lens, wherein intuition is understood as emergent, embodied, and environmentally responsive—shaped by socialization, experience, and institutional affordances (Aithal and Satpathy, 2024; Fine, 2017a).

By reframing intuition not as a deviation from rationality but as a valid, context-sensitive cognitive mode, this review contributes to ongoing efforts to decenter androcentric epistemologies—positioning female intuition as a legitimate form of situated leadership intelligence. The following discussion explores the broader implications of this synthesis—connecting the empirical domains with contemporary theoretical debates in economics, leadership studies, and feminist epistemology.

## 5 Discussion

The findings synthesized in this review respond directly to the four guiding questions by offering an integrative interpretation of how female intuition operates as a strategic epistemic resource within economic and organizational leadership. While the results were previously structured into five thematic domains, as detailed in Supplementary Table S4 (codebook); the present discussion revisits them through three overarching interpretive lenses:

1. The theoretical reconceptualization of intuition as an embodied and morally attuned form of cognition;
2. The contextual and intersectional dynamics that shape its expression and legitimacy; and
3. The methodological and translational implications for future research, leadership development, and institutional design.

Collectively, these lenses establish a multidimensional framework for critically understanding and applying the epistemic, cultural, and methodological significance of female intuition in leadership contexts.

This discussion does not treat intuition as a peripheral or anecdotal phenomenon but rather situates it at the core of adaptive leadership under uncertainty. Drawing upon 142 empirical studies, it advances a nuanced understanding of female intuition as a form of anticipatory intelligence—contextually enacted, ethically responsive, and shaped by the interplay of neurocognitive potential, social identity, and institutional affordances (Fine, 2017b; Ospina and Foldy, 2009). In doing so, it contributes to the redefinition of leadership intelligence through an epistemology grounded in embodiment, relationality, and cognitive plurality (Sinclair, 2011b; Isenman, 2018). For complementary treatments, see the chapters by Pretz (2011) (types of intuition), Hodgkinson and Sadler-Smith (2011) (methods), Klein (2011) (expert intuition/NDM), Duggan and Mason (2011) (strategic intuition), Guzak and Hargrove (2011) (ethical decision-making), and Bakken and Hærem (2011) (crisis decision-making) in Sinclair (2011b).

### 5.1 Reconceptualizing intuition in economic and leadership theory

The synthesis of the five domains supports a critical reformulation of dominant paradigms in cognitive and leadership theory. Drawing from interdisciplinary insights, this section distills how female intuition—understood as embodied, context-sensitive, and ethically attuned cognition—reconfigures prevailing assumptions about strategic decision-making.

First, the conceptual architecture of female intuition reveals a multidimensional construct comprising emotional attunement, contextual sensitivity, somatic cognition, and ethical reflexivity (Isenman, 2018; Sinclair and Ashkanasy, 2005). This view departs from simplified dual-process accounts—especially in popular treatments—that equate intuitive judgment with impulsivity or bias (Kahneman, 2011). In Epstein’s cognitive-experiential self-theory, the experiential mode is affect-laden and fast but not inherently impulsive (Epstein, 1998). It aligns with embodied, relational, and morally grounded perspectives (Fine, 2017a; Sinclair, 2011b). In this framing, female intuition is not merely fast cognition, but a situated epistemic capacity shaped by neurocognitive integration and gendered socialization.

Second, across domains such as risk perception and strategic foresight, empirical findings show that women’s intuitive reasoning does not displace analytical deliberation, but rather complements it (Delaney et al., 2014; Song, 2024). Far from reflecting static risk aversion, female intuition enables situationally attuned anticipation, ethical calibration, and responsive alignment with stakeholder needs under ambiguity (Downey et al., 2006; Ingersoll et al., 2023; Peni, 2014). This invites a move beyond essentialist narratives, toward a model of anticipatory intelligence embedded in relational ecologies.

Third, in the context of transformational leadership, female intuition plays a central role in fostering moral clarity, affective cohesion, and inclusive responsiveness (Eagly and Chin, 2010; Sinclair, 2011a). Empirical studies highlight how female leaders draw upon intuitive insight to detect latent tensions, nurture psychological safety, and enact ethically aligned change—especially in crisis or emotionally charged settings (De Blasio and Selva, 2020; Ghouse, 2023; Madison et al., 2022). Neuroscientific research supports this integration, noting that affective-intuitive processing is reinforced by interhemispheric coupling and oxytocin-modulated regulation (Aithal and Satpathy, 2024; Gur and Gur, 2017).

Fourth, in entrepreneurship and innovation, female intuition supports the alignment of strategy with stakeholder complexity, facilitating ethically grounded experimentation and adaptive decision-making (Goleman, 1995; Kolade et al., 2020). These capacities are not reducible to affective traits, but rather reflect the dynamic integration of emotional discernment, somatic awareness, and contextually situated cognition—particularly salient in resource-constrained or volatile environments (Madison et al., 2022; Song, 2024).

Fifth, the legitimacy of female intuition remains highly contingent across contexts (Ospina and Foldy, 2009; Visvanathan, 2007). In relational cultures such as East Asia or Latin America, intuition is often valorized. Conversely, hyper-rationalist institutions tend to marginalize intuitive insight unless reframed in analytical discourse (De Sousa Santos, 2014; Sinclair, 2007). These patterns reveal epistemic hierarchies and cognitive biases that continue to constrain the recognition of plural modes of reasoning (Fine, 2017a).

**Figure 7:**
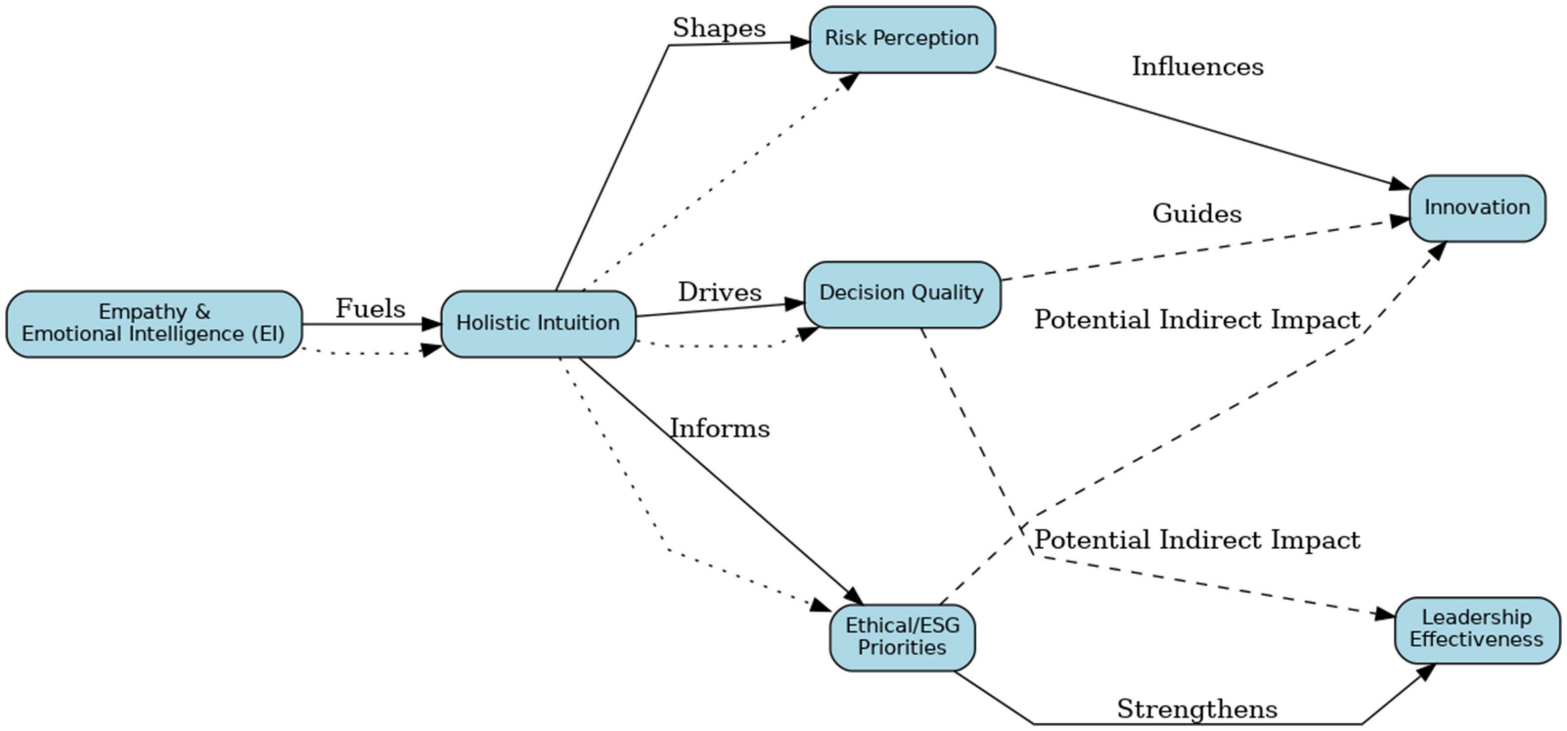
Holistic intuition pathways to innovation and ethical leadership.

Taken together, this reconceptualization contributes substantively to contemporary leadership theory. It challenges classical notions of disembodied rationality (Simon, 1955) by positioning intuition as a cognitively integrative and ethically responsive form of knowledge—especially crucial in high-stakes and ambiguous environments. The conceptual model developed integrates dual-process theory, feminist epistemology, embodied cognition, and neuroconstructivism (Hodgkinson and Sadler-Smith, 2017; Isenman, 2018) and is synthesized in Figure 7.

This integrative model finds further support in recent neuropsychological literature. Antonio Damasio’s (1996, 2021) somatic marker hypothesis emphasizes that intuitive judgments are grounded in pre-conscious somatic signals and emotional memory, particularly processed via the ventromedial prefrontal cortex (VMPFC). These findings reinforce the view that female intuition—characterized in this review by its affective, ethical, and embodied dimensions—has a plausible neurobiological basis.

Complementarily, Lieberman’s (2007) social neuroscience framework distinguishes reflexive from reflective processing systems, each with unique neural correlates. His work on the medial prefrontal cortex and anterior cingulate cortex aligns with the intuition pathways emphasized here, especially in emotionally complex interpersonal contexts. These insights further justify the integration of intuitive cognition into leadership theory.

Moreover, the manuscript benefits from clarifying the distinction between emotional affect and intuitive affect. While both are non-conscious and embodied, intuitive affect is directional, action-oriented, and often associated with tacit expertise, as noted by researchers such as Hogarth (2001) and Sadler-Smith (2008). This distinction is especially relevant when analyzing gendered patterns of intuitive engagement. Table 3 reinforces this point through comparative synthesis.

This conceptual framework illustrates how empathy and emotional intelligence fuel holistic intuition, which in turn drives risk perception, decision quality, and ethical and Environmental, Social, and Governance (ESG) priorities. These cognitive and ethical dimensions influence innovation outcomes and strengthen leadership effectiveness. The model reflects multidimensional pathways in which intuitive reasoning supports strategic agility, ethical foresight, and relational competence—particularly under conditions of ambiguity and volatility. Dashed lines indicate potential indirect pathways—i.e., effects operating through mediators (e.g., Decision Quality and/or ESG)—whereas dotted lines indicate emergent or hypothesized links that remain to be tested.

As visualized in this integrative framework, female intuition functions as a multidimensional conduit for ethical foresight, strategic innovation, and relational intelligence. It articulates how emotional attunement, somatic awareness, and moral reflexivity converge to produce adaptive leadership. The model also highlights the importance of intersectional moderators—such as gender, institutional culture, and neurobiological variation—in shaping the operationalization and legitimacy of intuitive reasoning across contexts.

This epistemic realignment extends Goleman’s (1995) emotional intelligence framework by embedding it within somatic discernment and ethical intentionality. It also reframes System 1 processing—not reducible to heuristics nor inherently biased, but fast, associative, and context-sensitive processes that can yield calibrated judgments when supported by experience and feedback, complementing System 2 deliberation under uncertainty (Evans and Stanovich, 2013; Kahneman and Klein, 2009).

In sum, female intuition is not an accessory to logic, but a strategic architecture of embodied knowing. This reframing aligns with emerging calls across leadership, organizational behavior, and cognitive science to recognize affective, embodied, and contextual intelligence as foundational to adaptive decision-making (Isenman, 2020; Fine, 2017a). Future leadership models must embrace this epistemic diversity to remain responsive to the moral complexity and cognitive plurality of contemporary organizational life.

### 5.2 Contextual and intersectional dynamics of female intuition

The legitimacy, expression, and institutional impact of female intuition cannot be understood in abstraction from the sociocultural and structural contexts in which it is enacted. As evidenced throughout this review, intuitive cognition is neither a fixed psychological trait nor a universally stable capacity. Rather, it constitutes a situated epistemic practice—modulated by the interaction of cultural norms, institutional logics, and neuropsychological dispositions (Lawrence and Suddaby, 2006; Crenshaw, 1991; Hodgkinson and Sadler-Smith, 2017; Sinclair, 2011b).

Cultural narratives decisively shape whether intuitive reasoning is validated, marginalized, or strategically reframed in leadership settings (Fine, 2017b; Ospina and Foldy, 2009). In Latin America, for instance, the cultural script of marianismo emphasizes emotional insight and moral responsibility among women leaders (Gil and Vásquez, 1996; Salazar Montoya and Kew, 2023), though often within restrictive frames of sacrifice and moral purity. In Confucian-influenced East Asia, intuition is linked to harmony and role-based ethics yet subordinated to hierarchical authority that may obscure women’s epistemic agency (Aithal and Satpathy, 2024; Heilemann and Faix, 2023). In Nordic countries, by contrast, intuitive leadership enjoys broader institutional legitimacy, supported by cultural commitments to emotional literacy and gender parity (Bas et al., 2022; Júliusdóttir et al., 2018; Larsson and Alvinius, 2020). Conversely, in Anglo-American corporate environments, affect-laden intuition is often viewed with skepticism unless translated into analytical or metric-compatible formats (Hallo and Nguyen, 2021; De Sousa Santos, 2014).

These cultural and institutional asymmetries reveal that the validation of female intuition is conditioned by the epistemological regimes governing each context—an evolution depicted in Supplementary Figure S5. Bureaucratic systems anchored in technocratic rationality, standardization, and auditability tend to marginalize embodied and affective cognition, particularly when expressed by women. Studies consistently report that female leaders feel compelled to post-rationalize intuitive decisions to fit dominant discourses, compromising both authenticity and epistemic autonomy (Croson and Gneezy, 2009; Delaney et al., 2014; Sinclair, 2007).

Nonetheless, these structural constraints are not immutable. As Amanda Sinclair (2007, 2011a) has argued, dismantling cognitive scripts that equate leadership with dispassionate rationality requires both epistemic pluralism and organizational models that embrace relational and embodied intelligence. Complementing this, Fine (2017a) critiques biological essentialism, emphasizing instead the neuroconstructive, socially situated nature of intuitive cognition. These insights support the claim that legitimizing female intuition is not merely a cultural accommodation—it is an epistemological imperative.

**Figure 8:**
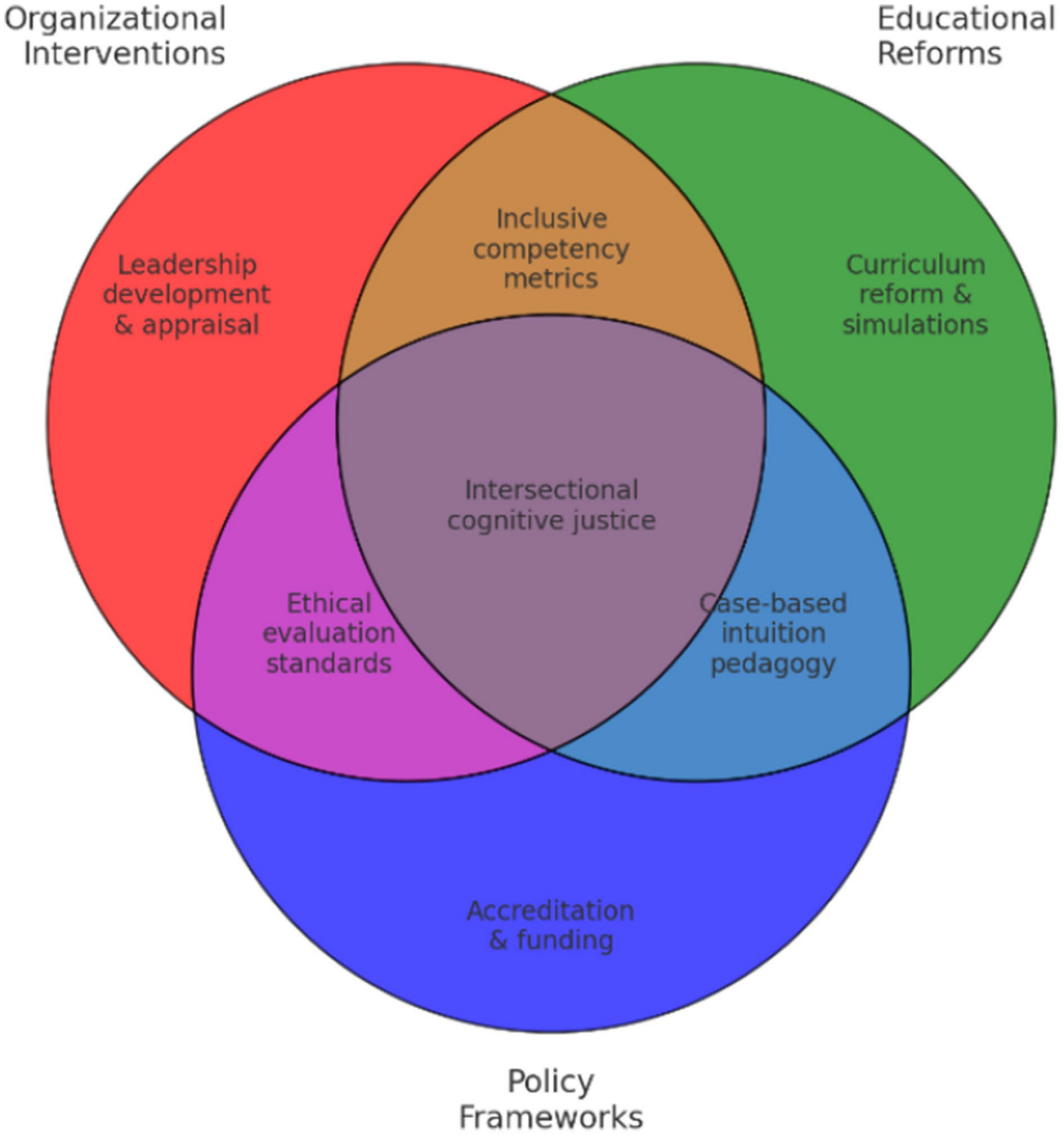
Intersections of organizational, educational, and policy interventions.

This conceptual realignment is synthesized visually in Figure 8, which maps how three institutional domains—organizational, educational, and policy-driven—interact to reinforce or suppress the epistemic legitimacy of intuitive reasoning in leadership contexts.

As illustrated, each domain activates distinct yet interdependent mechanisms that can either enable or constrain intuitive leadership. The discussion below elaborates these mechanisms, offering a roadmap for structural change.

At the organizational level, leadership development initiatives must move beyond the dominance of analytical paradigms. Empirical research indicates that experiential learning, somatic awareness training, and moral reflexivity enhance intuitive competence as detailed in Supplementary Figure S6, particularly for women navigating complex, ambiguous, or relationally charged environments (Delaney et al., 2014; Sadler-Smith, 2008). These approaches not only promote decision-making agility but also cultivate trust-based leadership and ethical foresight.

Evaluation systems likewise demand transformation. Traditional performance metrics often prioritize calculative efficiency, excluding dimensions such as emotional discernment, moral presence, and situational responsiveness (Sadler-Smith, 2008). Research confirms that women often face pressure to retroactively translate intuitive insights into acceptable rational discourse (Hallo and Nguyen, 2021; Isenman and Sinclair, forthcoming). Promoting epistemic pluralism requires redefining performance indicators to include embodied intelligence, contextual agility, and ethical reasoning.

At the policy level, regulatory and accrediting bodies can catalyze epistemic transformation by embedding intersectional and neurocognitive paradigms into leadership standards. Scholars such as Billing and Alvesson (2000) and De Sousa Santos (2014) call for policy frameworks that challenge the masculinized epistemologies underpinning leadership legitimacy. Initiatives grounded in feminist economics, neuroleadership, and cross-cultural strategy offer promising pathways for validating intuitive cognition as a credible leadership resource.

Educational institutions, especially in business and public policy, constitute critical levers for epistemic transformation. Studies demonstrate that curricula integrating embodied learning, narrative inquiry, and affective pedagogy enhance strategic foresight, moral clarity, and authentic leadership development (Durst et al., 2024). These findings underscore the translational potential of intuitive education—not only for individual leaders but for institutional cultures as a whole.

Crucially, all of these shifts must be anchored in intersectionality. Intuition is not legitimized equally across axes of race, class, age, or cultural background. Scholars such as Crenshaw (1991), Harding (1991), and De Sousa Santos (2014) remind us that knowledge is not neutral; intuitive competence is often delegitimized through racialized, gendered, and classed exclusions—particularly in data-centric, male-dominated, or Western institutions. Evaluation frameworks must thus remain sensitive to contextual complexity and actively dismantle epistemic hierarchies.

In sum, female intuition in leadership is not a reflection of essential gender traits, but an emergent product of embodied experience, neurocognitive plasticity, and sociocultural validation (Goleman, 1995; Isenman, 2018; Salazar Montoya and Kew, 2023). Its recognition requires a fundamental redefinition of epistemic legitimacy—one that values ethical complexity, affective intelligence, and plural ways of knowing (Harding, 1991; De Sousa Santos, 2014).

As demonstrated in the comparative matrix (Table 3), intuitive cognition is continuously negotiated—shaped by intersectional positionalities, power asymmetries, and institutional logics that determine what counts as valid knowledge (Crenshaw, 1991; Eagly and Chin, 2010). These findings reinforce the urgency of advancing leadership models that embrace cognitive diversity, and of developing methodologies that capture intuitive reasoning without reducing it to universalist or mechanistic paradigms (Isenman and Sinclair, forthcoming; Ospina and Foldy, 2009).

Ultimately, the legitimacy of female intuition is co-constructed at the intersection of cultural narratives, organizational structures, and neurobiological capacities. Future research must move beyond abstraction and investigate how intuitive cognition is enacted, contested, and institutionalized in diverse leadership ecologies, especially in non-Western and underrepresented contexts (Dane and Pratt, 2007; Ghouse, 2023). Doing so will not only enrich our theoretical understanding but also contribute to more inclusive, reflexive, and ethically grounded models of leadership for a plural and interdependent world.

### 5.3 Methodological horizons and future research agenda

This systematic review has consolidated a growing body of literature on female intuition in leadership, highlighting its emerging conceptual contours and exposing persistent methodological challenges that constrain scholarly advancement. While interdisciplinary approaches and neurocognitive frameworks have enriched the analytical landscape, further refinement is required to ensure empirical robustness, intersectional sensitivity, and contextual validity.

#### 5.3.1 Advances and ongoing limitations

Recent years have witnessed notable progress, particularly through mixed-methods designs that combine psychometric, behavioral, and contextual analysis to examine intuitive leadership. Studies such as Delaney et al. (2014) and Kolade et al. (2020) integrate qualitative insights with performance metrics, revealing how women draw upon embodied cognition to navigate complex organizational ecologies. Concurrently, neuroscientific research—as advanced by Aithal and Satpathy (2024) and Hallo and Nguyen (2021)—has expanded the analytical toolkit by linking intuitive reasoning with affective–prefrontal integration and oxytocin modulation.

Nevertheless, three methodological limitations remain salient.

First, there is a pervasive lack of construct clarity. Intuition is frequently conflated with emotion, instinct, or impulsivity, obscuring its distinctiveness as a form of fast, ethically attuned cognition (Dane and Pratt, 2007; Khatri and Ng, 2000). While typologies by Sinclair (2011b), Isenman (2018), and Sadler-Smith (2008) offer useful distinctions, their inconsistent application limits theoretical consolidation and hampers cross-study comparability (Dörfler and Ackermann, 2012).

Second, the role of gender remains undertheorized. Many studies treat it as a fixed demographic variable, failing to capture how intuition is shaped at the intersections of gender, race, class, culture, and institutional position (Brunetto et al., 2023; Eagly and Chin, 2010). Despite the relevance of intersectional epistemologies—as developed by Crenshaw (1991), Ospina and Foldy (2009), and Harding (2004)—they are rarely operationalized in empirical research, leaving unexamined how epistemic legitimacy is socially negotiated in diverse leadership environments (Atewologun, 2018).

Third, the geographic and linguistic scope of the literature remains narrowly defined. Most studies originate from North America and Western Europe, focusing disproportionately on Anglo-American corporate settings (Sinclair, 2020b). Valuable insights from Latin America, Africa, and Asia—particularly on relational intuition, communal resilience, and spiritual cognition—are largely absent (Alatas, 2006; Ghouse, 2023; Kolade et al., 2020; Sinclair, 2020b). The exclusion of gray literature and non-English sources further reinforces indexing biases that marginalize pluriversal epistemologies and embodied frameworks of knowing (De Sousa Santos, 2014; Mongeon and Paul-Hus, 2016).

These methodological limitations not only restrict analytical depth but also perpetuate epistemic exclusion (Fine, 2017b; Harding, 2004). Overcoming them demands a paradigmatic shift toward inclusive, context-sensitive, and pluralistic research practices. Of particular concern is the linguistic bias embedded in inclusion criteria. In the present review, the restriction to English-language publications—though necessary for bibliographic validation—limits the representation of non-Anglophone knowledge systems.

This is especially problematic in a field such as intuitive cognition, where affective constructs, cultural scripts, and vernacular epistemologies are deeply embedded in sociolinguistic environments (Alatas, 2006; Ospina and Foldy, 2009). Contributions from Latin American feminist theory, Afro-diasporic leadership traditions, and indigenous knowledge systems remain largely excluded—not due to conceptual irrelevance, but because of language-based gatekeeping in mainstream academic databases (Júliusdóttir et al., 2018; De Sousa Santos, 2014).

As De Sousa Santos (2014) and Harding (2004) have argued, such practices amount to epistemicide: the systematic erasure of alternative ways of knowing grounded in community, embodiment, and relational praxis.

To redress these exclusions, future reviews should adopt multilingual search protocols and expand data sources beyond dominant platforms like Scopus and Web of Science (Boell and Cecez-Kecmanovic, 2015). Regional databases such as SciELO, RedALyC, and African Journals Online must be systematically integrated. Search strategies should reflect the geographic, cultural, and linguistic scope of the inquiry. The formation of multilingual research teams or the establishment of cross-regional collaborations can enhance both the accuracy and ethical quality of evidence synthesis (Delaney et al., 2014). When direct inclusion is not feasible, translational approaches or consultation with local experts may approximate inclusion while preserving methodological integrity.

Ultimately, expanding linguistic scope is not merely a technical enhancement, it is a step toward epistemic justice. Given that female intuition is a culturally situated and discursively mediated construct, it cannot be fully understood within the confines of monolingual evidence. Integrating linguistic diversity enables a more equitable, accurate, and pluralistic representation of how intuitive cognition is enacted, perceived, and legitimized across global leadership contexts (Fine, 2017b; De Sousa Santos, 2014; Sinclair, 2011a).

#### 5.3.2 Priorities for future research and multilevel applications

To address the methodological and conceptual gaps identified, this review proposes three interrelated imperatives that can guide the advancement of research on female intuition in leadership:

1. Clarify constructs and diversify contexts

Future studies must refine the conceptualization of female intuition, clearly differentiating it from adjacent constructs such as empathy, emotionality, or prosociality (Hodgkinson and Sadler-Smith, 2017; Sinclair, 2011a). Developing multidimensional operational definitions grounded in cognitive science and feminist epistemology will enhance construct validity, enable theoretical coherence, and support the generalizability of empirical findings (Fine, 2017a; Salazar Montoya and Kew, 2023; Sinclair et al., 2024).

Cross-cultural research should explicitly test these refined constructs across diverse socio-institutional ecologies, identifying regional moderators of intuitive enactment and legitimacy (Cheng et al., 2024; Sitorus and Lusianah, 2023). This is particularly relevant in contexts where relational intelligence, embodied knowledge, and ethical discernment are culturally embedded yet institutionally underrecognized.

1. Adopt longitudinal and neurophenomenological methods

To trace the development and variability of intuitive competence over time, future studies should incorporate longitudinal designs—especially during critical leadership moments such as organizational crises, strategic transitions, or innovation cycles (Ingersoll et al., 2023; Madison et al., 2022). These designs will allow researchers to map how intuitive reasoning evolves and interacts with experience, affect regulation, and moral learning.

Complementing this, neurophenomenological approaches—such as combining fMRI or EEG data with reflective interviews or embodied narrative tracking—offer promising avenues for examining how somatic cues correlate with moral discernment, affective awareness, and relational attunement in real time (Aithal and Satpathy, 2024; Gur and Gur, 2017; Isenman, 2020). Such methods move beyond behaviorist reductionism to capture the embodied and temporally situated nature of intuitive cognition.

1. Embed intersectionality as an epistemological lens

Intersectionality must evolve from a demographic descriptor to a foundational epistemological stance. Rather than isolating gender as a binary or additive variable, researchers should investigate how multiple axes of identity—such as race, class, age, ethnicity, and institutional position—shape the enactment and legitimacy of intuitive cognition (Atewologun, 2018; Crenshaw, 1991; Ospina and Foldy, 2009).

Qualitative strategies such as narrative inquiry, embedded ethnographies, and participatory design can capture how diverse women negotiate the use and recognition of intuition within structurally constrained and affectively charged environments (DeVault and Gross, 2012; Thrasher et al., 2023). These methodologies are particularly apt for mapping the interplay between affective labor, epistemic bias, and institutional power in leadership settings.

To translate these priorities into multilevel applications, this review proposes a three-tiered framework that integrates the theoretical, practical, and institutional dimensions of future research and policy:

1. Theoretical level

Refine existing dual-process models to incorporate somatic awareness, moral intentionality, and identity-based cognition. Theoretical integrations should draw from feminist theory, neuroleadership, and moral psychology to construct models of intuitive reasoning that reflect both biological grounding and social embeddedness (Epstein, 2010; Hodgkinson and Sadler-Smith, 2017; Isenman, 2018; Sinclair, 2011b). These models must transcend individualist or decontextualized views of cognition and instead position female intuition as a relational and situational form of strategic intelligence.

1. Practical level

Design leadership training programs that cultivate intuitive competence through structured somatic practices, guided moral reflection, and scenario-based learning. Educational initiatives—especially in fields such as public policy, education, and business management—should adopt pedagogies of embodiment that validate affective reasoning as both teachable and ethically consequential (Bas et al., 2022; Delaney et al., 2014). This reframing enables leaders to integrate intuitive awareness into complex decision-making without relying solely on analytic or instrumental rationality.

1. Institutional level

Reform leadership evaluation systems to recognize non-analytical competencies such as ethical foresight, affective discernment, and contextual agility. Accrediting bodies, funding agencies, and policy institutions should develop inclusive metrics that legitimize epistemic diversity in strategic reasoning and organizational judgment (Eagly and Chin, 2010; Ospina and Foldy, 2009; Sinclair and Ashkanasy, 2005). Rather than privileging abstract calculation, these reforms would foreground the value of embodied ethics and relational insight.

These recommendations seek to advance a reframing of female intuition not as an anecdotal or incidental trait, but as a strategic cognitive resource—theoretically grounded, empirically emergent, and pragmatically relevant for complex leadership ecosystems. By aligning methodological rigor with epistemological inclusion, future research can move beyond reductive binaries and capture the full complexity of how women lead, decide, and transform their organizations through intuitive intelligence.

### 5.4 Epistemic reframing and strategic conclusion

When conceptualized as a somatically grounded, ethically attuned, and contextually embedded mode of cognition, female intuition emerges as a vital dimension of adaptive leadership—particularly in environments characterized by uncertainty, relational complexity, and moral ambiguity (Aithal and Satpathy, 2024; Isenman, 2018; Salazar Montoya and Kew, 2023). Drawing from a multi-disciplinary evidence base, this review reframes intuitive competence not as a deviation from rationality, but as a culturally situated epistemology embedded in gendered socialization, neurocognitive potential, and institutional structure.

At its core, female intuition—as theorized throughout this synthesis—is not an anecdotal or residual form of knowing. It constitutes a multidimensional cognitive architecture that integrates emotional intelligence, somatic awareness, ethical reflexivity, and contextual discernment. It arises from the interplay between neurobiological integration, social conditioning, and positionality within institutional ecologies. Neuroscientific findings underscore the relevance of interhemispheric connectivity and oxytocin-mediated affective regulation as enablers of anticipatory, relationally grounded cognition (Aithal and Satpathy, 2024; Gur and Gur, 2017; Isenman, 2018), while feminist and embodied cognition theories frame this intelligence within practices of care, reflexivity, and relational agency (Fine, 2017b; Harding, 1991; Salazar Montoya and Kew, 2023; Sinclair, 2011b).

The evidence demonstrates that female leaders frequently rely on intuition to navigate complex decision environments—whether recalibrating risk, aligning with stakeholder needs, fostering innovation, or leading during institutional crises (Delaney et al., 2014; Kolade et al., 2020). Yet its legitimacy remains contested, especially when evaluated through frameworks grounded in disembodied rationalism and instrumental logic. However, this review reveals that female intuition functions as a strategic epistemic capacity—responsive to context, grounded in relational foresight, and aligned with ethical discernment.

This synthesis yields three primary insights:

1. It consolidates female intuition as a legitimate and teachable cognitive capacity, bridging neurobiological mechanisms with cultural scripts and institutional validation (Bas et al., 2022; Sinclair and Ashkanasy, 2005).
2. It extends dual-process models by integrating somatic knowledge, moral alignment, and gendered experience into strategic cognition—moving beyond dichotomies of logic versus emotion (Fine, 2017b; Goleman, 1995; Isenman, 2020).
3. It advances an epistemological shift from static typologies toward embodied, relational, and ethically grounded models of leadership, as proposed by feminist organizational theory and neuroconstructivist paradigms (Harding, 1991; Ospina and Foldy, 2009; Sinclair, 2011b).

To translate these insights into culturally responsive frameworks, Table 5 presents a comparative cultural matrix that maps epistemic scripts—drawing on the data summarized in Supplementary Table S6—together with validation norms and leadership archetypes that condition the expression and legitimacy of female intuition across regions (De Blasio and Selva, 2020; De Sousa Santos, 2014).

**Table 5:** Comparative cultural framework.

Region	Epistemic scripts	Validation mechanisms	Leadership models
Nordic Europe	Emotional literacy, consensus, inclusivity	Transformational legitimacy, psychological safety	Inclusive–empathic leadership
East Asia	Harmony, communal responsibility, hierarchy	Role-based respect, relational accountability	Relational–deferential leadership
Latin America	Marianismo, moral authority, affective ethics	Symbolic credibility, ethical alignment	Ethico-relational leadership
Anglo-American Context	Rationalism, data primacy, instrumental logic	Metrics, KPIs, strategic justification	Transactional–performative leadership
Sub-Saharan Africa	Resilience narratives, communal wisdom	Community validation, adaptive storytelling	Contextual–resilience leadership

This framework emphasizes the situated nature of intuitive cognition and the epistemic hierarchies that structure its recognition. Methodologically, it demonstrates the value of triangulating narrative synthesis, critical appraisal, and bibliometric mapping to illuminate not only consensus but also silences and exclusions within the field.

Looking forward, the challenge is not merely to document intuition, but to transform the evaluative frameworks through which it is interpreted, cultivated, and legitimized. Institutions must move beyond reductionist epistemologies and adopt cognitive architectures grounded in plural rationalities, ethical depth, and embodied intelligence (Harding, 2004; De Sousa Santos, 2014; Sinclair and Ashkanasy, 2005). This reframing demands more than methodological inclusivity; it requires a redefinition of epistemic legitimacy itself—one that acknowledges intuition as a form of anticipatory intelligence, rooted in affective discernment, moral presence, and situational attunement (Fine, 2017b; Isenman, 2018).

Ultimately, this review affirms that female intuition is not a deviation from logic—it is a different logic: one that is relational, contextually anchored, and ethically responsive. It fuses emotional acuity with somatic resonance and ethical clarity, offering a leadership capacity particularly attuned to uncertainty, interdependence, and systemic fragility (Delaney et al., 2014; Khatri and Ng, 2000). In such a world, embracing this epistemic modality is not only a matter of epistemic justice—it is a strategic imperative for cultivating inclusive, adaptive, and morally grounded decision-making (Crenshaw, 1991; Ospina and Foldy, 2009).

### 5.5 Policy and organizational implications

The insights generated by this review hold not only theoretical and methodological relevance, but also significant implications for public policy, institutional design, and leadership development. If female intuition is to be fully integrated into governance frameworks, policy must move beyond rhetorical commitments to diversity and enact structural mechanisms that recognize and valorize embodied, relational, and ethically attuned ways of knowing (Billing and Alvesson, 2000; Harding, 2004; De Sousa Santos, 2014).

First, leadership development programs—particularly in the public sector—should explicitly incorporate indicators of intuitive competence as summarized in Supplementary Table S5. A growing body of evidence indicates that embodied awareness and structured reflection enhance moral discernment and decision-making under complexity (Bas et al., 2022; Isenman, 2018; Sadler-Smith, 2008). For instance, the UN Women Training Centre’s “Leadership, Empowerment, and Accountability” initiative integrates emotional intelligence and gender-responsive ethics into civil servant formation, providing a scalable model for embedding intuitive cognition into national leadership academies (Eagly and Chin, 2010; UN Women Training Centre, 2016).

Second, evaluation systems in public administration must evolve to include relational and qualitative indicators that reflect moral clarity, affective insight, and cognitive diversity. Traditional performance metrics—focused on calculative efficiency—tend to marginalize the contributions of intuitive leadership (Goleman, 1995; Sinclair and Ashkanasy, 2005). In response, the OECD’s Public Leadership Framework advocates embedding adaptive, inclusive, and ethically grounded competencies into civil service assessments to enhance trust and responsiveness in volatile environments (OECD, 2020; OECD, 2021).

Third, gender equity policies must address not only representation but also the epistemic dimensions of inclusion. Increasing the presence of women in leadership roles is necessary but insufficient if non-analytical, embodied reasoning remains illegible within prevailing evaluative logics (Billing and Alvesson, 2000; Fine, 2017b). Ospina and Foldy (2009) argue that inclusive leadership requires not only demographic diversity but also the legitimacy of alternative cognitive modes, including intuitive intelligence—especially in institutions where dominant rationalities systematically exclude embodied cognition (Crenshaw, 1991; Harding, 2004).

Fourth, cross-sector collaborations—between ministries of education, public service commissions, and academic institutions—can facilitate the development of diploma programs in Embodied Leadership and Ethical Intelligence. Empirical research from Latin America and Southeast Asia suggests that pedagogical models integrating neuroleadership, contextual ethics, and affective reflexivity foster female leadership in public service (Acevedo-Duque et al., 2021). These initiatives can be institutionalized through regional platforms such as the Escuela del Gobierno Abierto of CLAD (2023), which promotes ethical, inclusive leadership at the municipal and state level.

Fifth, international organizations can act as epistemic catalysts by incorporating intuitive intelligence into global frameworks for leadership and governance. Bodies such as the OECD’s Observatory of Public Sector Innovation (OPSI) and UNDP’s Gender Equality Seal have advanced indicators that recognize emotional literacy, ethical foresight, and relational judgment as critical leadership competencies (Eagly and Carli, 2007; OPSI, 2021; UNDP, 2021). These standards should inform funding criteria, accreditation protocols, and policy guidelines for leadership training and organizational capacity building.

In sum, intuition is not merely an individual trait—it is a socially cultivated, neurocognitively enabled, and institutionally conditioned capacity. Public policy must therefore construct the epistemic infrastructure necessary for its recognition, development, and application (De Sousa Santos, 2014; Sinclair, 2011b). Doing so will not only deepen gender equity in leadership, but also enhance moral agility, strategic foresight, and adaptive responsiveness in complex governance environments. In this context, valuing female intuition is not simply a symbolic gesture—it is a structural imperative for ethical and resilient leadership.

## 6 Conclusion

This systematic review reconceptualizes female intuition as a multidimensional, embodied, and contextually enacted mode of strategic cognition—positioned at the intersection of gender, neurocognitive integration, and leadership praxis. Synthesizing 142 peer-reviewed studies across disciplines and geographies, the findings challenge reductive interpretations of intuition as emotional reactivity, reframing it as a legitimate epistemic modality grounded in ethical discernment, affective attunement, and somatic intelligence (Aithal and Satpathy, 2024; Hallo and Nguyen, 2021).

Empirically, female intuition emerges as a pivotal resource for decision-making under uncertainty, transformational leadership, innovation, and stakeholder engagement. Rather than opposing analytical reasoning, it complements and enhances it—supporting relationally grounded, ethically responsive, and anticipatory judgment in complex environments (Delaney et al., 2014; Khushk et al., 2022; Madison et al., 2022). These findings redefine intuition not as a gendered trait, but as a context-sensitive capacity shaped by sociocultural scripts, institutional logics, and intersectional positionalities (Crenshaw, 1991; Ospina and Foldy, 2009).

Theoretically, this review contributes to the reframing of leadership cognition by integrating embodied awareness and moral intentionality into mainstream decision-making models. Dual-process accounts do not inherently privilege deliberation; rather, it is prevalent social and organizational thinking that tends to privilege deliberative logic in evaluation and reporting. Against that backdrop, this synthesis elevates intuitive discernment as a strategic faculty—situated, relational, and ethically aligned. Female intuition is thus repositioned as a socially embedded and morally attuned form of knowing—distinct from emotional impulsivity, and modulated by gender norms, leadership ecologies, and institutional structures (Hodgkinson and Sadler-Smith, 2017; Salazar Montoya and Kew, 2023; Sinclair, 2011a).

Practically, these insights underscore the urgency of transforming how institutions train, evaluate, and empower decision-makers. Leadership development, performance evaluation, and policy frameworks must include tools that validate intuitive intelligence—especially in its affective, ethical, and contextual dimensions (Folbre, 2001; Sadler-Smith, 2008; Sinclair and Ashkanasy, 2005). Doing so demands a systemic commitment to intersectional cognitive justice, ensuring that historically marginalized forms of reasoning are not merely accommodated but recognized as central to organizational adaptability (Billing and Alvesson, 2000; Harding, 2004).

Methodologically, the field requires more multimodal, neurophenomenological, and cross-cultural research designs. Future studies should move beyond binary gender framings to explore how intuitive cognition manifests across neurodiverse profiles, relational identities, and structural environments (Atewologun, 2018; Thrasher et al., 2023). The integration of behavioral analysis, neurocognitive imaging, and narrative inquiry will be vital for capturing the embodied and relational dimensions of intuition *in situ* (Isenman, 2018; Ospina and Foldy, 2009).

At the neurobiological level, this review cautions against essentialist readings of intuition. While pathways involving oxytocin and estrogen have been linked to affective reasoning, the evidence emphasizes neuroplasticity, social learning, and institutional feedback loops as more significant drivers of intuitive competence (Gur and Gur, 2017; Joel and Vikhanski, 2019; Sinclair, 2011b). Cognition, as these findings affirm, is emergent—not predetermined, but relationally shaped through dynamic interaction between biology, identity, and cultural encoding.

This epistemic reframing becomes especially salient in light of two global transformations: the rise of artificial intelligence and the rupture of post-pandemic leadership paradigms. As algorithmic governance advances, the risk of eclipsing human intuition intensifies. Yet intuitive reasoning—particularly as enacted by women—offers an indispensable counterbalance: it embodies ethical foresight, situational discernment, and affective resonance, all of which remain beyond the reach of computational logic (Goleman, 1995; Kandasamy, 2024; Khatri and Ng, 2000). The COVID-19 crisis further revealed the limitations of technocratic leadership, highlighting the value of relational, contextual, and embodied cognition in navigating moral uncertainty and institutional fragility (Eagly and Chin, 2010; Fine, 2017b; De Sousa Santos, 2014).

Repositioning female intuition at the heart of leadership theory affirms a broader cognitive shift: one that legitimizes somatic knowledge, ethical reflexivity, and relational intelligence as core to decision-making in complex systems. Recognizing intuition as a situated and morally responsive form of intelligence invites institutions to rethink how they define excellence, legitimacy, and strategic foresight (Ospina and Foldy, 2009; Sinclair, 2011a). In a world increasingly shaped by abstraction and algorithmic bias, restoring the legitimacy of embodied cognition is not just a matter of epistemic justice—it is a strategic imperative for building inclusive, resilient, and ethically grounded leadership.

## Data Availability

The original contributions presented in the study are included in the article/Supplementary material, further inquiries can be directed to the corresponding author/s.
