# Supplementary material for: Female intuition in economics and conscious leadership: a comprehensive integrative review of conceptual foundations, cultural moderators, and future directions
Source: Front Sociol. 2025 Sep 30;10:1560090. doi: 10.3389/fsoc.2025.1560090 (PMC12519457; doi:10.3389/fsoc.2025.1560090)
Supplement: Supplementary file 1 [file Supplementary_file_1.pdf]

Supplementary Material

Female Intuition in Economics and Conscious Leadership: Supplementary Tables and Figures

Table S1. Summary and quality assessment of the 142 studies included in the review (abbreviated view).
The complete filterable matrix—including all column definitions—is provided in ***Supplementary File S7 (Excel)****.*

Study (Author, Year)	Design / Method	Appraisal Tool (Version)	Screened ✓	Critical Items Met / N (%)	Non-critical Items Met / N (%)	Risk of Bias	Overall Confidence †	Included in Synthesis
**Muzayanah & Anggraeni (2023)**	Qualitative multi-case study	CASP 2018	Yes	9 / 10 (90%)	N/A	Low	★★★★ (High)	Yes
**Xiao et al. (2024)**	Quantitative study	MMAT 2018	Yes	3 / 5 (60%)	4 / 5 (80%)	Moderate	★★★ (Moderate)	Yes
**Role of Female Leadership (2022)**	Mixed-methods systematic review	MMAT 2018	Yes	5 / 7 (71%)	10 / 14 (71%)	Moderate	★★★ (Moderate)	Yes
**Khagan & Redondo-Sama (2024)**	Systematic review	AMSTAR 2	Yes	6 / 7 (86%)	9 / 14 (64%)	Low	★★★★ (High)	Yes
**Luoma & Martela (2021)**	Conceptual / Theoretical	N/A	Yes	N/A	N/A	N/A	N/A	No (Theory)

Note. Quality ratings were assigned with three validated instruments: Critical Appraisal Skills Programme (CASP) for qualitative studies, Mixed Methods Appraisal Tool (MMAT 2018) for mixed-methods and quantitative studies, and AMSTAR 2 for systematic reviews. "Critical items" denote domains essential for internal validity—CASP items 2–9; MMAT items 1–5; AMSTAR 2 items 2, 4, 7, 9, 11, 13, 15. Overall confidence follows AMSTAR 2 grading: ★★★★ High, ★★★ Moderate, ★★ Low. Percentages indicate the proportion of critical items met (n/N). N/A = Not applicable (this item was not evaluated for the study).

Table S2. Search Protocol and Inclusion Criteria Summary

Component	Details
**Databases**	Scopus, Web of Science, PsycINFO, PubMed, EconLit
**Search period**	February 3–5, 2025
**Search terms**	“intuition”, “female leadership”, “gender”, “decision-making”, “embodied cognition”, “emotional intelligence”
**Boolean logic used**	AND/OR combinations
**Controlled vocabulary**	MeSH (PubMed), APA Thesaurus (PsycINFO), Keywords Plus (Web of Science)
**Inclusion criteria**	Peer-reviewed, 2000–2024, gender lens, leadership or decision relevance
**Exclusion criteria**	Non-peer-reviewed, lack of gender analysis, purely technical or statistical studies
**Languages included**	English only
**Snowball sampling**	Backward citation tracking and expert recommendation (22 studies)

Note. Full Boolean strings, date limits and language filters are listed. Database abbreviations: Sc = Scopus; WoS = Web of Science; PM = PubMed; PI = PsycINFO; EL = EconLit.

Table S3. Quality Appraisal Summary Framework

Appraisal Tool	Purpose	Criteria Evaluated	Source
**CASP (2018)**	Evaluate qualitative studies	Credibility, relevance, reflexivity, ethical integrity	Critical Appraisal Skills Programme
**MMAT (2018)**	Appraise mixed-methods designs	Sampling, measurement, integration of methods, validity	Hong et al., 2018
**AMSTAR 2**	Assess quality of systematic reviews	Search strategy, bias control, heterogeneity, reporting	Shea et al., 2017

Note. Quality ratings were assigned with three validated instruments: Critical Appraisal Skills Programme (CASP) for qualitative studies, Mixed Methods Appraisal Tool (MMAT 2018) for mixed-methods studies, and AMSTAR 2 for systematic reviews. “Critical items” refer to tool-specific domains essential for internal validity—CASP items 2-9; MMAT items 1-5; AMSTAR 2 items 2, 4, 7, 9, 11, 13 and 15. Overall confidence follows AMSTAR 2 grading: *** High, ** Moderate, * Low. Percentages reflect the proportion of critical items met (n/N).

Table S4. Thematic Clusters and Codes Derived from Narrative Synthesis

Thematic Domain	Code	Representative Quote	Studies Referenced
**Conceptualization of Female Intuition**	Embodied Cognition	'Somatic awareness informs ethical foresight in female leaders'	Sinclair, 2011b; Isenman, 2018
**Risk Perception and Decision-Making**	Ethical Anticipation	'Female leaders anticipate stakeholder needs under uncertainty'	Delaney et al., 2014; Downey et al., 2006
**Transformational Leadership**	Relational Leadership	'Women intuitively sense team dynamics and adjust responses'	Ghouse, 2023; Madison et al., 2022
**Innovation and Strategic Agility**	Contextual Sensitivity	'Women in high-volatility contexts pivot using intuitive foresight'	Khushk et al., 2022; Kolade et al., 2019
**Cultural and Structural Moderators**	Epistemic Legitimacy	'Intuition is often dismissed unless reframed analytically'	Santos, 2014; Sinclair, 2007

Note. Codes derived via reflexive thematic analysis. Brief definitions provided in the 'Codebook' column.

Table S5. Indicators of Intuitive Competence in Leadership Assessment Frameworks

Indicator	Operational Definition	Assessment Modality	Supporting Literature
**Ethical Foresight**	Anticipates moral dilemmas and evaluates long-term consequences of decisions	Narrative simulations, ethical scenario analysis	Sadler-Smith, 2008; Fine, 2017
**Contextual Agility**	Adapts cognitive and relational strategies to shifting environmental cues	Behavioral observation, 360° feedback	Goleman, 1995; Sinclair & Ashkanasy, 2005
**Affective Resonance**	Accurately reads and responds to emotional dynamics within teams	Emotional intelligence assessments, team interviews	Eagly & Chin, 2010; Ghouse, 2023
**Somatic Awareness**	Uses bodily signals as cues for decision-making under uncertainty	Somatic reflection journals, biofeedback training	Isenman, 2018; Aithal & Satpathy, 2024
**Relational Accountability**	Demonstrates commitment to trust-building, care ethics, and stakeholder engagement	Leadership narratives, stakeholder interviews	Ospina & Foldy, 2009; Khushk et al., 2022

Note. Indicators grouped by cognitive (C), affective (A) and ethical (E) domains; asterisk (*) denotes items validated in ≥ 2 empirical studies.

Table S6. Comparative Framework of Epistemic Scripts

Epistemic Dimensions	Western	East Asian	Latin American	Sub-Saharan African
**Cultural schema**	Individualism	Collectivism, harmony	Familism, Catholic ethos	Communalism (*Ubuntu*), spiritual relationality
**Validation mechanism**	Logical coherence	Contextual sensitivity	Ethical alignment	Oral consensus, ancestral wisdom, communal resonance
**Leadership model**	Transformational, goal-driven	Relational-participatory	Popular-democratic, ethical	Community-rooted, elder-guided, spiritually anchored
**Contextual notes**	Rationalist merit, autonomy	Culturally encoded deference	Colonial residues, affective navigation of power	Wisdom through intergenerational narratives, moral storytelling, intuition as ancestral attunement

Note. Matrix compares dominant, relational and embodied epistemic scripts across ontology, axiology and method.

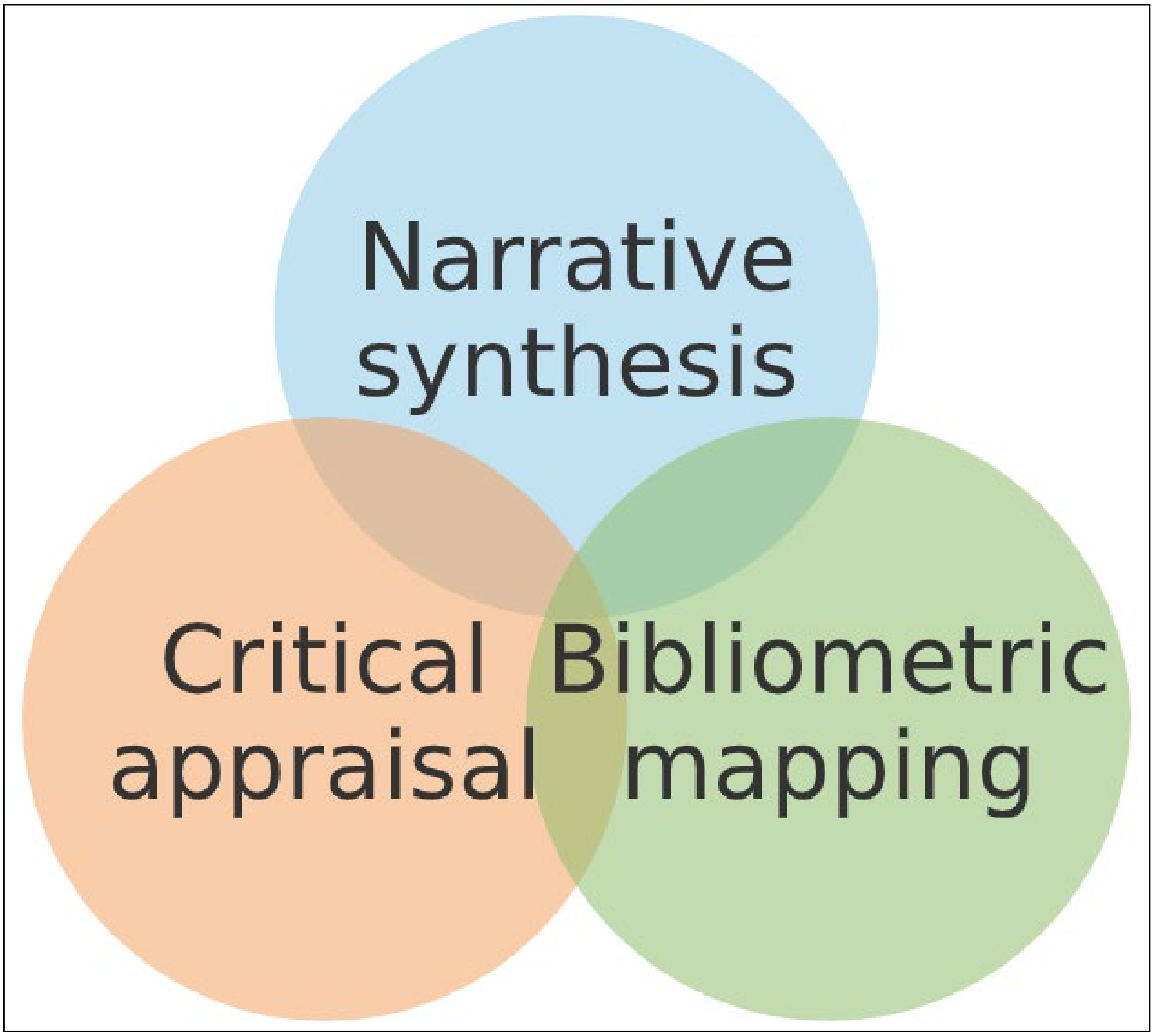

Figure S1. Triangulated analysis workflow

Schematic overview of the three-tier synthesis used in the review—narrative synthesis, reflexive thematic coding and VOSviewer-based bibliometric mapping—showing how each stream iteratively informed the others and converged on the final thematic architecture.

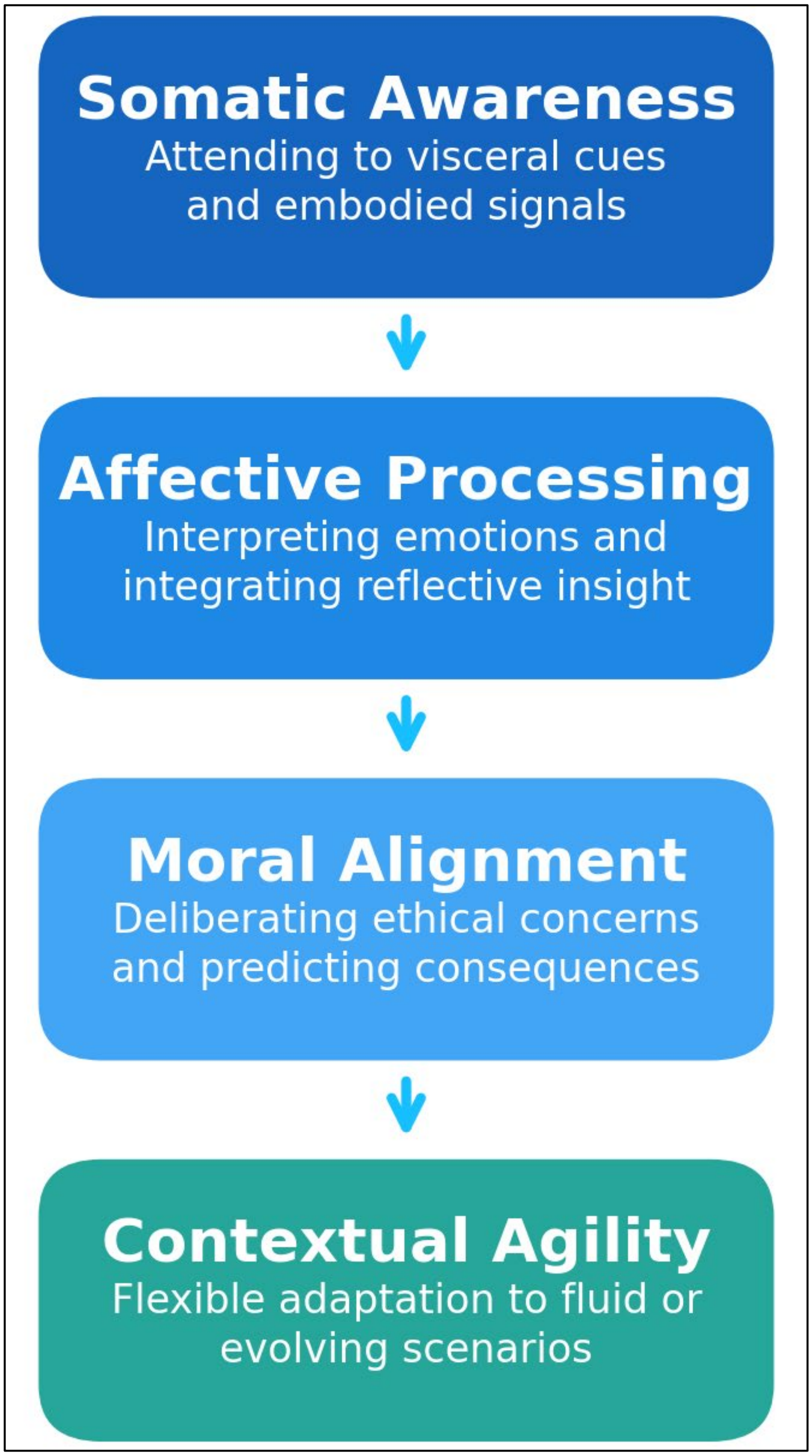

Figure S2. Intuitive-Leadership Process Model.

Sequential pathway illustrating how *somatic awareness* initiates intuitive cognition, progressing through affective processing and moral alignment to culminate in contextual agility. The model distils the micro-processes by which female leaders translate embodied cues into adaptive strategic action.

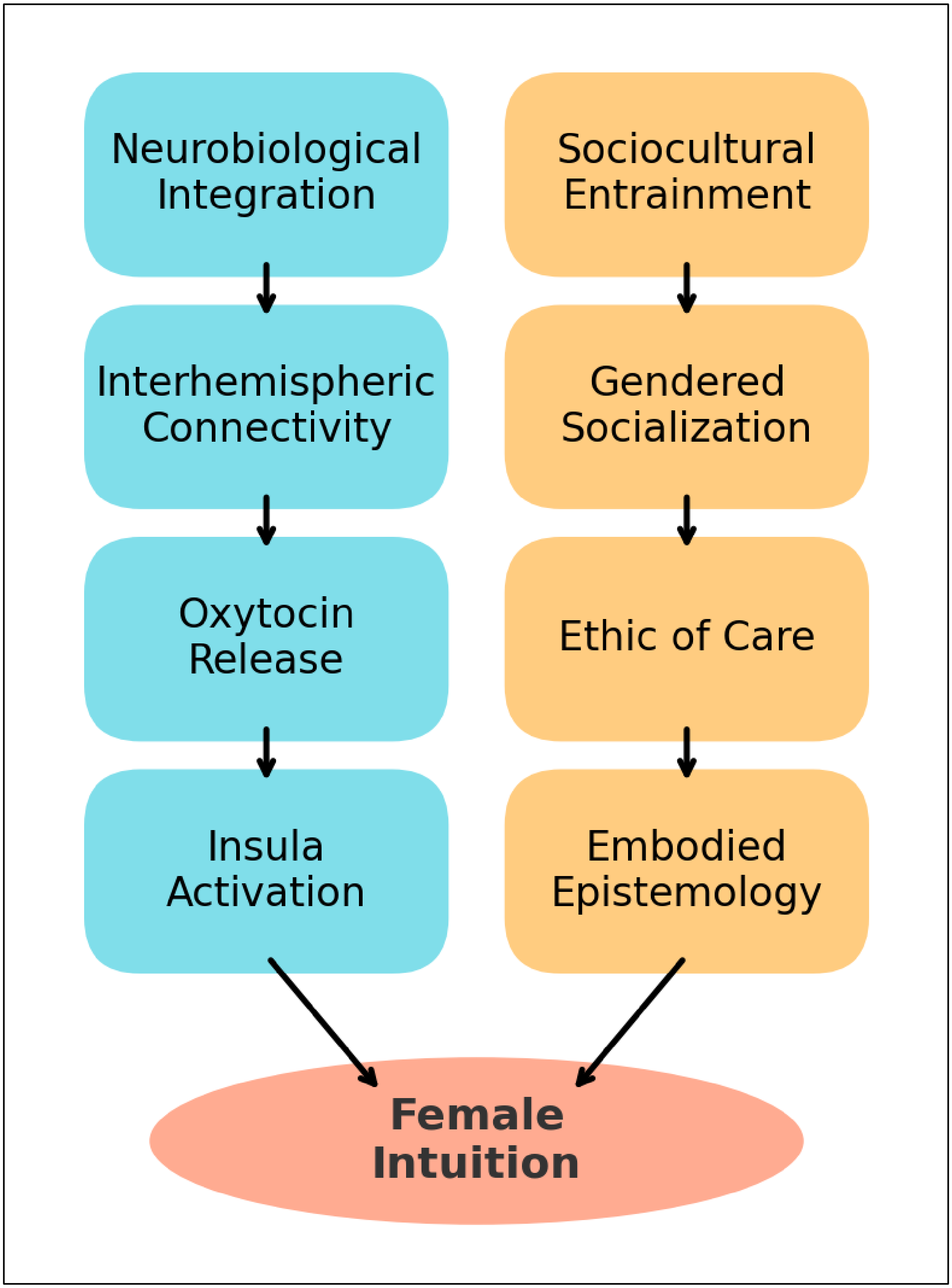

Figure S3. Converging Neurocognitive and Social Pathways

Parallel tracks depict neurobiological mechanisms (inter-hemispheric connectivity, oxytocin-mediated insula activation) and sociocultural mechanisms (gendered socialization, ethic of care, embodied epistemology) that fuse into the multidimensional construct of female intuition.

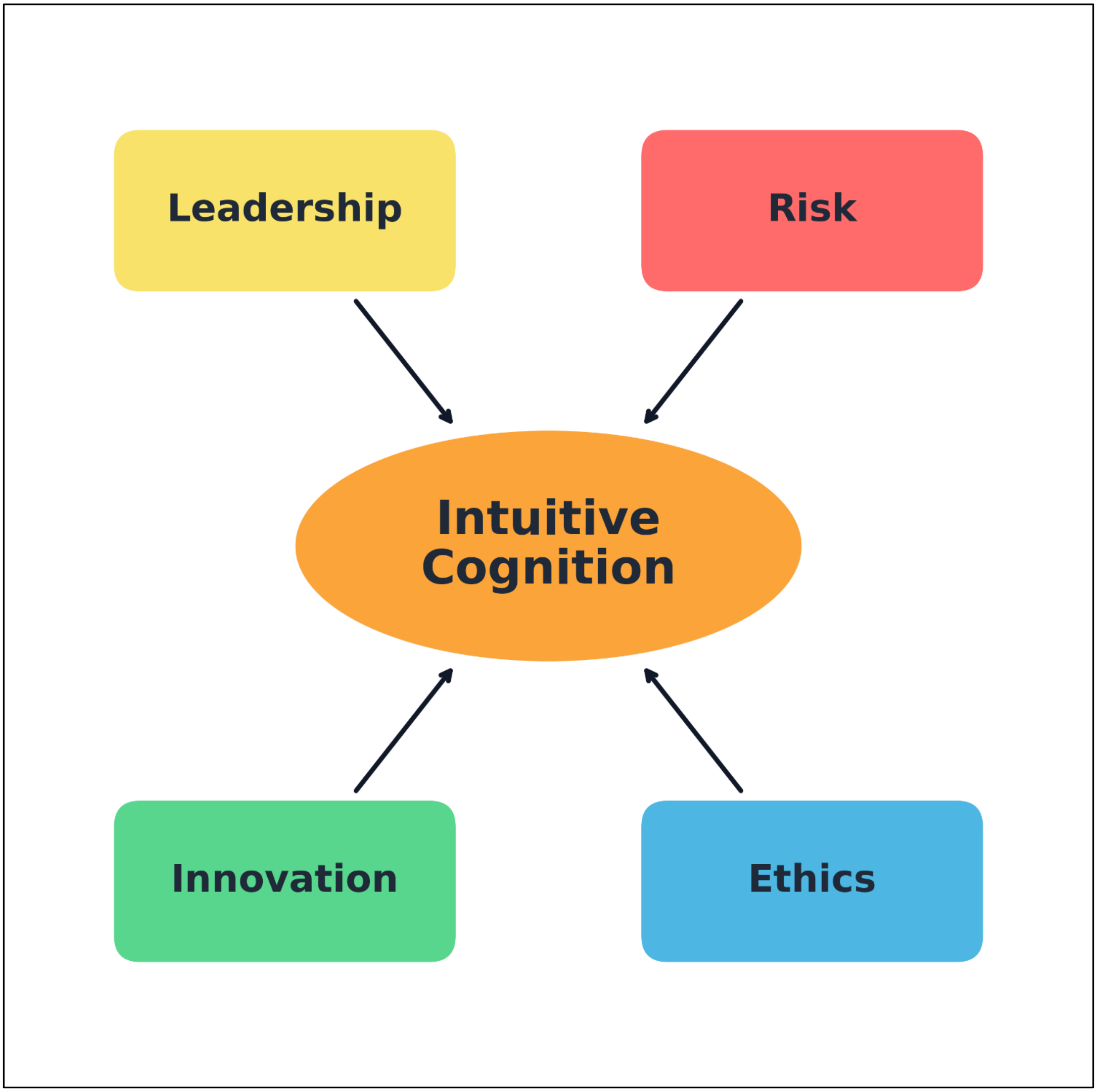

Figure S4. Dimensions Contributing to Intuitive Cognition.

Conceptual map showing how leadership, risk, innovation, and ethics contribute to the development of intuitive cognition.

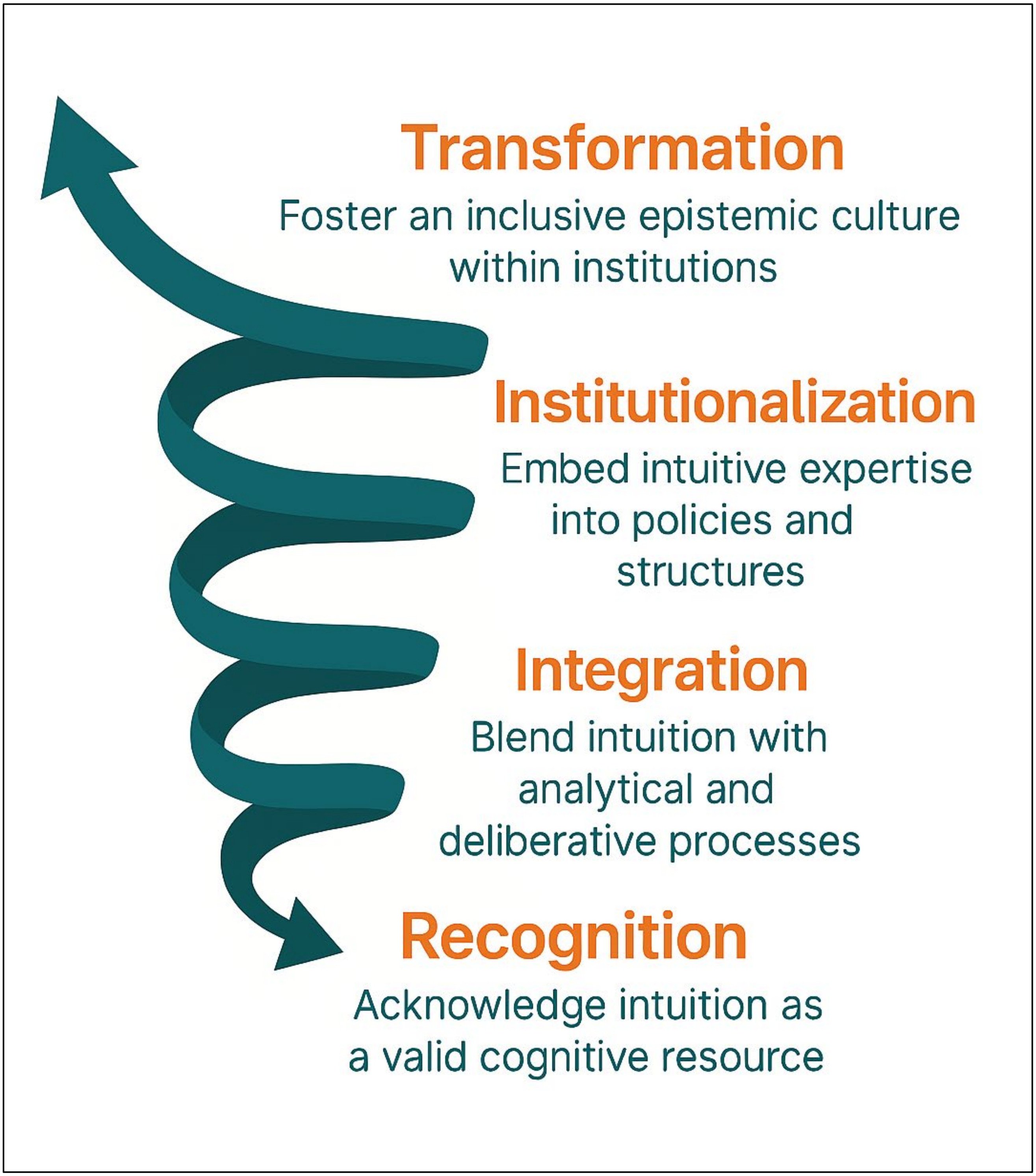

Figure S5. Epistemic Inclusion Spiral: From Recognition to Institutionalization.

Spiral diagram tracing four escalating stages—recognition, integration, validation and institutionalization—through which embodied and affective ways of knowing transition from marginal status to accepted organizational practice.

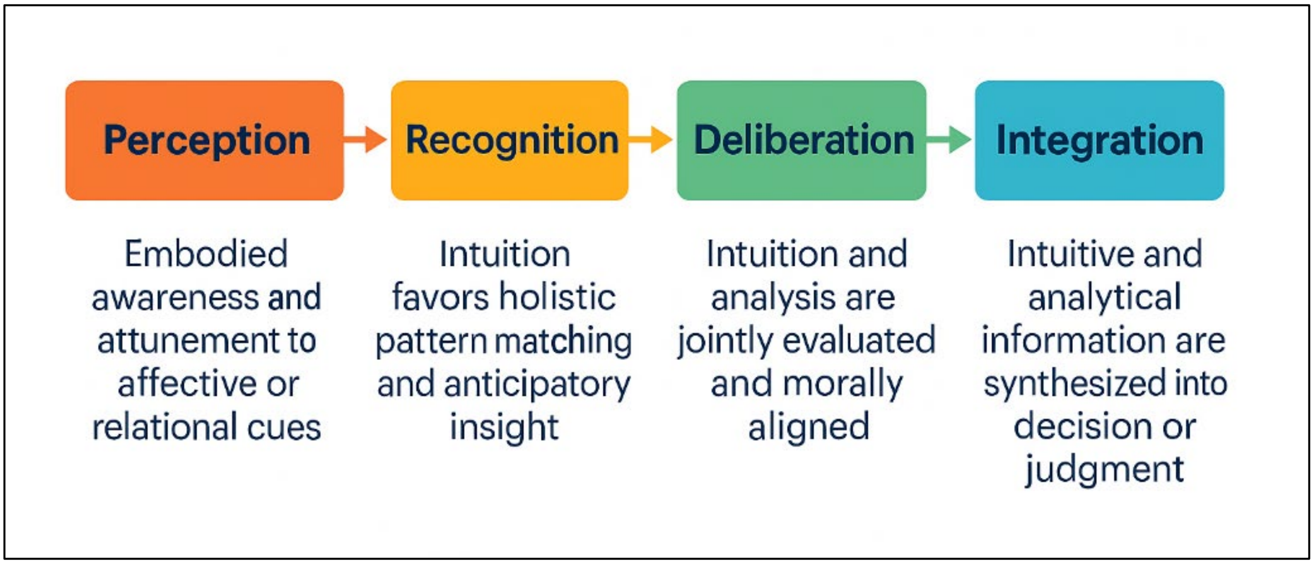

Figure S6. Proposed Four-Stage Model of Female Intuition in Leadership

Conceptual model detailing a cyclical progression—anticipatory sensing, ethical framing, relational calibration and strategic enactment—that links intuitive judgment to transformational outcomes across iterative decision episodes.
